# Camonsertib in DNA damage response-deficient advanced solid tumors: phase 1 trial results

**DOI:** 10.1038/s41591-023-02399-0

**Published:** 2023-06-05

**Authors:** Timothy A. Yap, Elisa Fontana, Elizabeth K. Lee, David R. Spigel, Martin Højgaard, Stephanie Lheureux, Niharika B. Mettu, Benedito A. Carneiro, Louise Carter, Ruth Plummer, Gregory M. Cote, Funda Meric-Bernstam, Joseph O’Connell, Joseph D. Schonhoft, Marisa Wainszelbaum, Adrian J. Fretland, Peter Manley, Yi Xu, Danielle Ulanet, Victoria Rimkunas, Mike Zinda, Maria Koehler, Ian M. Silverman, Jorge S. Reis-Filho, Ezra Rosen

**Affiliations:** 1 Department of Investigational Cancer Therapeutics, The University of Texas MD Anderson Cancer Center, Houston, TX USA; 2 Sarah Cannon Research Institute UK, London, UK; 3 Department of Medical Oncology, Dana-Farber Cancer Institute, Boston, MA USA; 4 Sarah Cannon Research Institute/Tennessee Oncology, Nashville, TN USA; 5 Department of Oncology, Rigshospitalet, Copenhagen, Denmark; 6 Princess Margaret Cancer Centre, Toronto, ON Canada; 7 Department of Medical Oncology, Duke University, Durham, NC USA; 8 Legorreta Cancer Center at Brown University and Lifespan Cancer Institute, Division of Hematology/Oncology, Department of Medicine, Warren Alpert Medical School, Brown University, Providence, RI USA; 9 Division of Cancer Sciences, University of Manchester and the Christie NHS Foundation Trust, Manchester, UK; 10 Newcastle University and Newcastle Hospitals NHS Foundation Trust, Northern Centre for Cancer Care, Newcastle-upon-Tyne, UK; 11 Massachusetts General Hospital Cancer Center, Boston, MA USA; 12 Repare Therapeutics, Cambridge, MA USA; 13 Department of Pathology, Memorial Sloan Kettering Cancer Center, New York, NY USA; 14 Department of Medical Oncology, Memorial Sloan Kettering Cancer Center, New York, NY USA

**Keywords:** Translational research, Predictive markers, Tumour biomarkers, Cancer genetics

## Abstract

Predictive biomarkers of response are essential to effectively guide targeted cancer treatment. Ataxia telangiectasia and Rad3-related kinase inhibitors (ATRi) have been shown to be synthetic lethal with loss of function (LOF) of ataxia telangiectasia-mutated (ATM) kinase, and preclinical studies have identified ATRi-sensitizing alterations in other DNA damage response (DDR) genes. Here we report the results from module 1 of an ongoing phase 1 trial of the ATRi camonsertib (RP-3500) in 120 patients with advanced solid tumors harboring LOF alterations in DDR genes, predicted by chemogenomic CRISPR screens to sensitize tumors to ATRi. Primary objectives were to determine safety and propose a recommended phase 2 dose (RP2D). Secondary objectives were to assess preliminary anti-tumor activity, to characterize camonsertib pharmacokinetics and relationship with pharmacodynamic biomarkers and to evaluate methods for detecting ATRi-sensitizing biomarkers. Camonsertib was well tolerated; anemia was the most common drug-related toxicity (32% grade 3). Preliminary RP2D was 160 mg weekly on days 1–3. Overall clinical response, clinical benefit and molecular response rates across tumor and molecular subtypes in patients who received biologically effective doses of camonsertib (>100 mg d^−1^) were 13% (13/99), 43% (43/99) and 43% (27/63), respectively. Clinical benefit was highest in ovarian cancer, in tumors with biallelic LOF alterations and in patients with molecular responses. ClinicalTrials.gov registration: NCT04497116.

## Abstract (web summary)

In patients with DNA damage response-deficient solid tumors, the oral ATR kinase inhibitor camonsertib was well tolerated; a recommended phase 2 dose was identified; and encouraging anti-tumor activity was observed, with the highest response rate observed in patients with ovarian cancer.

## Main

The DNA damage response (DDR) is indispensable for the maintenance of genomic integrity and cell survival. Loss of specific components of the DDR machinery results in distinct forms of genomic instability^1^. The ataxia telangiectasia and Rad3-related (ATR) kinase plays an integral role in the DDR by triggering a cascade of events in response to DNA damage and replication stress^2,3^. Targeting DDR defects through synthetic lethality is a clinically validated approach for the treatment of cancer^4–6^. This approach is exemplified by poly adenosine diphosphate-ribose polymerase (PARP) inhibitors, which have received regulatory approval to treat patients with multiple tumor types with *BRCA1* or *BRCA2* (*BRCA1/2*) loss-of-function (LOF) mutations and other selected alterations in different settings.

In preclinical and early clinical studies, ATR inhibition has been shown to be synthetically lethal with LOF of the ataxia telangiectasia-mutated (ATM) kinase^7,8^. Although early clinical studies investigating ATR inhibition in tumors harboring *ATM* mutations or lacking ATM protein expression have shown preliminary signals of anti-tumor activity, the optimal method for identifying *ATM* LOF in a broader population remains to be established. We hypothesize that the accurate diagnosis and treatment of *ATM* LOF tumors requires the determination of allelic status (biallelic versus non-biallelic) and the exclusion of *ATM* LOF alterations stemming from clonal hematopoiesis. Furthermore, we hypothesize that ATR inhibition results in anti-tumor activity in DDR alterations beyond *ATM*, such as *BRCA1/2* and others. Specifically, the clinical activity of ATR inhibition in PARP inhibitor (PARPi)-resistant tumors, including cancers with *BRCA1/2* reversion mutations, has not been reported. We investigated if ATR inhibition could be beneficial for these patients and in other critical areas of unmet clinical need.

**Fig. 1:**
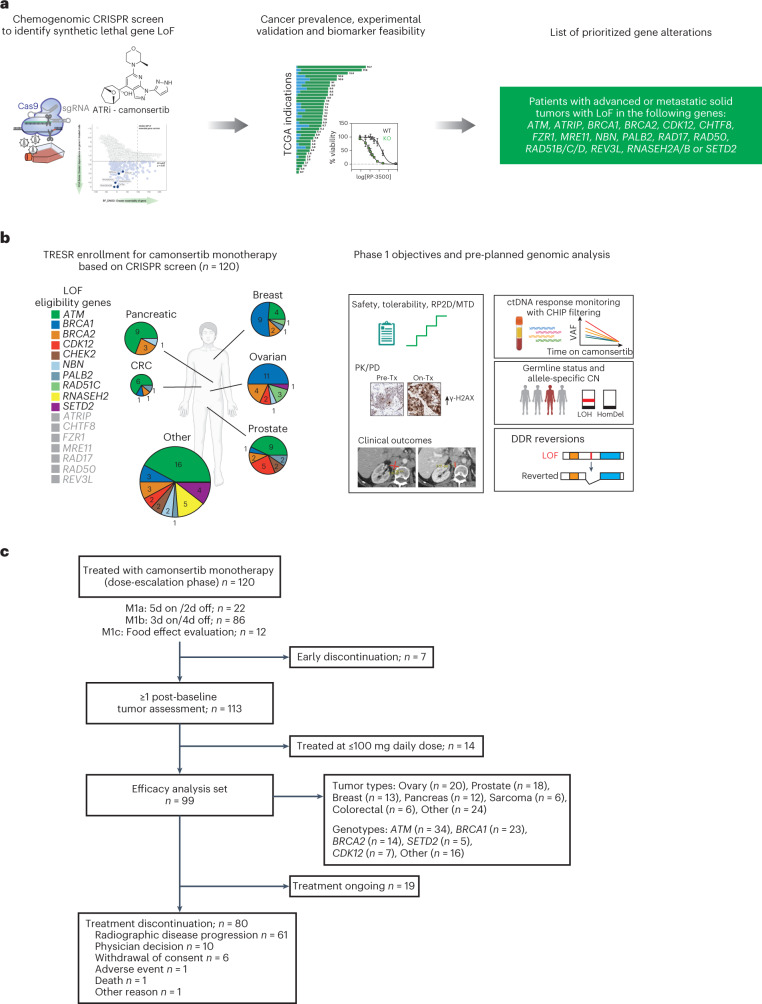
Overview of the TRESR trial: a CRISPR–Cas9 chemogenomic-informed clinical trial. **a**, SNIPRx CRISPR–Cas9-enabled chemogenomic screen to identify ATRi-sensitizing and synthetic lethal alterations for patient selection. **b**, Patient enrollment by gene and tumor type and overview of pre-planned analyses, which included (1) clinical endpoints; (2) PK in plasma and PD in pre-treatment and on-treatment biopsies; (3) hypothesis-generating genomic analyses, such as the assessment of allelic status (that is, biallelic versus non-biallelic alterations) and somatic versus germline status; and (4) analysis of longitudinal ctDNA as an early marker of camonsertib activity. **c**, CONSORT diagram of TRESR monotherapy patient populations. Patients enrolled in M1c received a single dose on day 3 in the fed state and continued from day 1 (fasted state) on either the 5/2 (*n* = 3) or 3/4 (*n* = 9) schedule. 3/4, 3 d on, 4 d off; 5/2, 5 d on, 2 d off; CN, copy number; CRC, colorectal cancer; HomDel, homozygous deletion; M, module; TCGA, The Cancer Genome Atlas; Tx, therapy.

Multiple ATR inhibitor (ATRi)-sensitizing cancer alterations have been proposed by means of RNA interference-enabled or CRISPR–Cas9-enabled forward chemogenomic screening^9–13^. We used these chemogenomic CRISPR-enabled screen datasets, together with internal and published preclinical validation data, to identify ATRi-sensitizing DDR alterations as the rational basis for patient selection for treatment with camonsertib (RP-3500) (Methods and Fig. 1a)^10,13–19^.

Here we report results of a phase 1 clinical trial (Treatment Enabled by SNIPRx (SyNthetic Lethal Interactions for Precision Therapeutics platform) (TRESR)) of camonsertib in patients with DDR biomarker-selected advanced solid tumors (NCT04497116). The primary objectives were to assess safety and tolerability and to propose a recommended phase 2 dose (RP2D). Secondary and exploratory objectives were to determine anti-tumor activity, pharmacokinetics (PK), pharmacodynamics (PD), predictive biomarkers and circulating tumor DNA (ctDNA) dynamics. A key requirement for trial eligibility was the presence of an ATRi-sensitizing gene alteration (LOF of *ATM*, *ATRIP*, *BRCA1*, *BRCA2*, *CDK12*, *CHTF8*, *FZR1*, *MRE11*, *NBN*, *PALB2*, *RAD17*, *RAD50*, *RAD51B/C/D*, *REV3L*, *RNASEH2A*, *RNASEH2B* or *SETD2*; Fig. 1a). Several of the eligibility genes, such as *SETD2* and *RNASEH2B*, are distinct from the canonical homologous recombination repair (HRR) genes associated with sensitivity to PARPi. Pre-planned translational analyses were designed to (1) define the context in which solid tumors are sensitive to camonsertib, including tumor type and genomic profile; (2) test the hypothesis that biallelic LOF of the gene alteration would enrich for clinical benefit to camonsertib; and (3) define if early ctDNA dynamics predict clinical outcomes to camonsertib.

## Results

### Trial design and characteristics of camonsertib

The TRESR trial was designed to assess the use of preclinically identified and validated ATRi-sensitizing alterations as the basis for patient selection (Fig. 1a,b). A suite of integrated clinicogenomic analyses was incorporated into the clinical trial design to identify patients with advanced solid tumors harboring prospectively identified molecular alterations for whom treatment with camonsertib is feasible and effective (Fig. 1b,c and Supplementary Fig. 1).

We describe the results from 120 patients enrolled in module 1 of TRESR treated with camonsertib monotherapy. This module is closed to enrollment (enrollment in the gemcitabine combination in TRESR remains ongoing). Key inclusion criteria were age of ≥18 years at the time of consent; Eastern Cooperative Oncology Group (ECOG) performance status score of 0 or 1; histologically confirmed solid tumor resistant or refractory to standard treatment and/or intolerance to standard therapy; and the presence of a deleterious or likely deleterious gene alteration in the specified set of genes expected to sensitize tumors to ATR inhibition. Key exclusion criteria included treatment with chemotherapy; small molecule or biologic anti-cancer therapy within 14 d before first dose of the study drug; or prior therapy with an ATR or DNA-dependent protein kinase (DNA-PK) inhibitor.

Baseline characteristics of the enrolled patients are shown in Table 1. The most common tumor types were ovarian (*n* = 22; 18.3%), castration-resistant prostate cancer (CRPC) (*n* = 21; 17.5%), breast (*n* = 17; 14.2%) and pancreatic (*n* = 13; 10.8%). The most frequent genetic alterations of those enrolled were in *ATM* (*n* = 44; 36.7%), *BRCA1* (*n* = 25; 20.8%), *BRCA2* (*n* = 15; 12.5%) and *CDK12* (*n* = 9; 7.5%) (Table 1 and Fig. 1b,c). Central next-generation sequencing (NGS) testing by the Synthetic Lethal Interactions for Precision Diagnostics panel (SNiPDx)^20^ or whole-genome sequencing (WGS) detected the enrollment alteration in 96.5% (83/86) of cases with sufficient material.

**Table 1:** Patient disposition and pre-treatment biomarkers

	All patients (*n* = 120)
Sex, *n* (%)^a^	
Male	49 (40.8)
Female	71 (59.2)
Age (years), median (range)	63 (30–77)
≥65 years, *n* (%)	54 (45.0)
ECOG performance status, *n* (%)	
0	56 (46.7)
1	64 (53.3)
Lines of prior systemic therapy, *n* (%)	
≤3	69 (57.5)
4 or more	51 (42.5)
Prior platinum, *n* (%)	81 (67.5)
Prior PARP inhibitor, *n* (%)	39 (32.5)
Prior PD-(L)1 inhibitor, *n* (%)	29 (24.2)
Tumor type, *n* (%)	
Ovarian	22 (18.3)
Prostate	21 (17.5)
Breast	17 (14.2)
Pancreatic	13 (10.8)
Sarcoma	7 (5.8)
Other^b^	40 (33.3)
Enrollment gene, *n*	
*ATM*	44
*BRCA1*	25
*BRCA2*	15
*CDK12*	9
RNAseH2	5
*PALB2*	5
*SETD2*	5
Other^c^	12
Enrollment test type, *n*	
Tissue NGS	71
Germline	29
ctDNA	13
IHC (RNAseH2/ATM)	7 (5/2)
Central confirmation, *n*	
Tested	109
Confirmed	83
Unconfirmed	26
Not detected	3
Poor tissue quality	10
Alteration not covered	13
Origin, *n*	
Germline	55
Somatic	42
Undetermined (IHC enrollment)	23 (7)
Allelic status, *n*	
Biallelic	57
Non-biallelic	27
Monoallelic	20
CHIP	3
No loss	3
Subclonal	1
Unknown	36
Indeterminate	26
Not tested	9
Inconsistent	1
Reversion, *n*	
Detected	10

^a^Sex (at birth) as reported by the patient to the study site. ^b^Other tumor types included ampullary, appendix, bile duct, endometrial, gastrointestinal, head and neck squamous cell carcinoma, lung, melanoma, mesothelioma, sarcoma and skin. ^c^Other enrollment genes included *NBN* (*n* = 4), *RAD51B/C* (*n* = 4) and *CHEK2* (*n* = 4).

PD-1, programmed cell death protein 1; PD-L1, programmed death ligand 1.

### Primary endpoints

The starting dose of camonsertib was 5 mg administered on a 5-d-on, 2-d-off (5/2) schedule given once daily (QD) in 21-d cycles. Dose escalation proceeded until a total daily dose of 160 mg was achieved. Per protocol, based on emerging safety, PK and PD data, an alternative schedule of 3 d on, 4 d off (3/4) was then started at a daily dose of 120 mg and escalated to 200 mg, and 160 mg QD (3/4) of camonsertib was proposed as the preliminary RP2D based on long-term (>6 weeks) safety, tolerability, PK and PD data. The dose-limiting toxicity (DLT) rate at the proposed preliminary RP2D was 8% (2/25). All DLTs comprised hematologic toxicities (Table 2).

**Table 2:** DLTs and TRAEs

DLTs (all treated patients, DLT evaluable^a^ (*n* = 92))											
	5/2 schedule					3/4 schedule			3/4 schedule, 2/1w		
	5–80 mg QD (*n* = 5)	40 mg BID (*n* = 1)	100 mg QD (*n* = 4)	120 mg QD (*n* = 4)	160 mg QD (*n* = 3)	120 mg QD (*n* = 17)	60 mg BID (*n* = 3)	160 mg QD (*n* = 25)	160 mg QD (*n* = 25)	200 mg QD (*n* = 5)	Total (*n* = 92)^a^
Any DLT event, *n* (%)	0	0	2 (50.0)	0	0	1 (5.9)	1 (33.3)	2 (8.0)	2 (8.0)	1 (20.0)	9 (9.8)
Anemia	0	0	1 (25.0)	0	0	1 (5.9)	1 (33.3)	2 (8.0)	1 (4.0)	1 (20.0)	7 (7.6)
Platelet count decreased	0	0	1 (25.0)	0	0	0	0	0	0	1 (20.0)	2 (2.2)
Febrile neutropenia	0	0	0	0	0	0	0	0	0	1 (20.0)	1 (1.1)
Neutrophil count decreased	0	0	0	0	0	0	0	0	1 (4.0)	0	1 (1.1)

TRAEs^b^ (all treated patients (*n* = 120))						
	5/2 schedule (*n* = 25)			3/4 schedule (*n* = 95)		
	All grades	Grade 3	Grade 4	All grades	Grade 3	Grade 4
Any TRAE, *n* (%)	22 (88.0)	14 (56.0)	1 (4.0)	82 (86.3)	30 (31.6)	4 (4.2)
Anemia	20 (80.0)	13 (52.0)	0	61 (64.2)	25 (26.3)	0
Fatigue	7 (28.0)	1 (4.0)	0	26 (27.4)	2 (2.1)	0
Neutrophil count decreased/neutropenia	6 (24.0)	3 (12.0)	1 (4.0)	25 (26.3)	10 (10.5)	3 (3.2)
Platelet count decreased/thrombocytopenia	7 (28.0)	2 (8.0)	1 (4.0)	22 (23.2)	6 (6.3)	1 (1.1)
Nausea	3 (12.0)	0	0	23 (24.2)	0	0
Decreased appetite	4 (16.0)	0	0	14 (14.7)	0	0
Diarrhea	0	0	0	13 (13.7)	0	0
Vomiting	3 (12.0)	0	0	9 (9.5)	0	0
White blood cell count decreased	1 (4.0)	0	0	11 (11.6)	4 (4.2)	0
Dyspnea	5 (20.0)	0	0	6 (6.3)	0	0
Dysgeusia	1 (4.0)	0	0	5 (5.3)	0	0

^a^Patients enrolled in M1c (food effect study; *n* = 12) were not considered as part of the DLT-evaluable population. ^b^Occurring in ≥5% of the total treated population.

2/1w, 2 weeks on/1 week off; 3/4, 3 d on/4 d off; 5/2, 5 d on/2 d off; BID, twice daily; M, module.

Anemia was the most common treatment-related adverse event (TRAE) of any grade (67.5% of patients across all dose levels/schedules). Clinically significant anemia requiring dose modifications or transfusions typically manifested after the DLT period, following a slow decline in hemoglobin, and was the most common reason for dose holds and modifications. Overall, only 1/120 (0.8%) patients discontinued camonsertib (after four cycles) due to treatment-related grade 3 anemia. Anemia was more frequent in patients treated on the 5/2 schedule (52% grade 3, 80% all grades) than those on the 3/4 schedule (26% grade 3, 64% all grades); no grade 4 anemia was observed. Other common TRAEs on the 3/4 schedule (*n* = 95) were fatigue (27.4% overall, 2.1% grade 3), neutropenia (26.3% overall, 10.5% grade 3 and 3.2% grade 4), nausea (24.2% overall, all grade <3) and thrombocytopenia (23.2% overall, 6.3% grade 3 and 1.1% grade 4). Other non-hematologic toxicities were less common and low grade. The frequencies of TRAEs and treatment-emergent adverse events (TEAEs) are presented in Table 2 and Extended Data Table 1, respectively. No effect on QT interval was observed (Supplementary Fig. 2).

### Secondary endpoints

**Extended Data Fig. 1:**
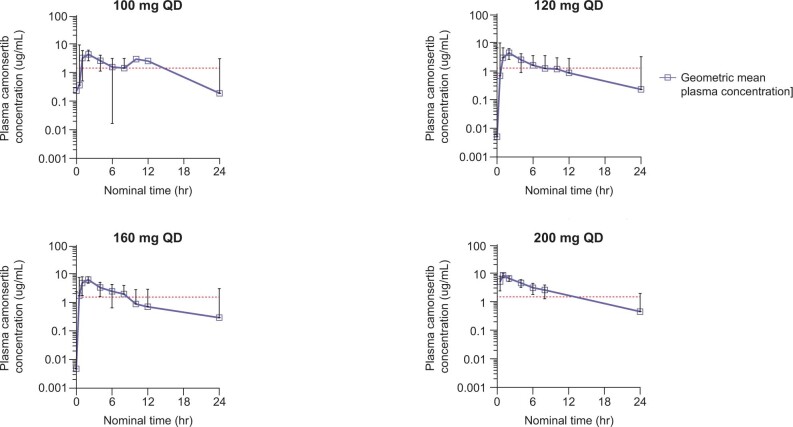
Pharmacokinetics of camonsertib at pharmacologically active dose levels. Geometric mean plasma concentration time profiles of camonsertib on cycle 1 day 1 are plotted at specified dose levels of 100 mg QD (*n* = 8 patients), 120 mg QD (*n* = 31 patients), 160 mg QD (*n* = 63 patients), or 200 mg (*n* = 5 patients). Error bars represent geometric standard deviation. The red dashed line represents the pre-clinical in vivo tumor pCHK1 IC_80_, which is the target for pharmacological activity (~10–12 hours coverage). Note that not every patient was evaluated at every timepoint. IC_80_, 80% inhibitory concentration; hr, hours; QD, once daily.

The PK profile of camonsertib exhibited low intra-patient and inter-patient variability, and a 5.8-h median half-life across all QD dose levels (interquartile range (IQR) 4.8–7.1); no accumulation after repeated dosing, was observed. Over the dose range of 5–200 mg QD, increases in maximum observed plasma concentration (C_max_) and area under the concentration time curve (AUC) were linear (Extended Data Fig. 1), whereas the median time to reach C_max_ (T_max_) was 1–2 h (Supplementary Table 1). Plasma exposures at doses >100 mg QD achieved predicted efficacious exposures based on preclinical models (efficacy associated with free concentrations of camonsertib above the in vivo tumor IC_80_ for pCHK1 inhibition for 10–12 h) (ref. ^14^). Twelve patients were enrolled in a food effect submodule (module 1c (M1c)). Administration of a high-fat, high-calorie meal resulted in modest PK changes, not anticipated to meaningfully impact the clinical safety or tolerability of camonsertib (Supplementary Fig. 3).

**Extended Data Fig. 2:**
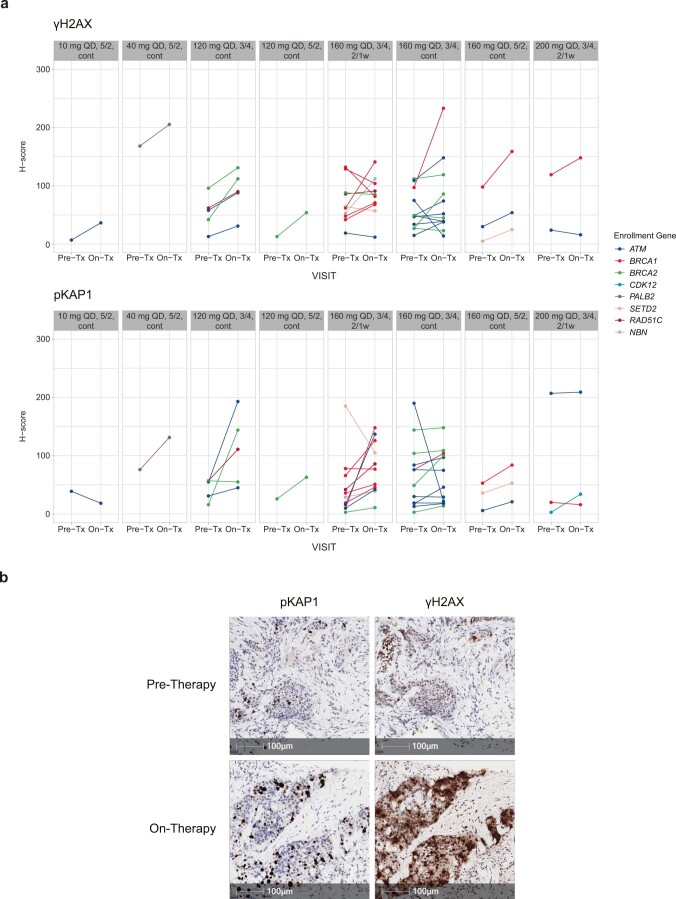
Pharmacodynamic biomarkers of ATR inhibition. (**a**) yH2AX (top) and pKAP1 (bottom) changes in H-score with camonsertib treatment from baseline to cycle 2 day 10 in patients treated with camonsertib at the indicated dose and schedules. (**b**) Representative histology micrographs from pre- and on-treatment biopsies taken 3 days prior to treatment initiation and at 30 days on-treatment from a patient with ER+ breast cancer treated with 160 mg QD camonsertib on the 3/4 continuous schedule. 2/1w, 2 weeks on, 1 week off; 3/4, 3 days on, 4 days off; 5/2, 5 days on, 2 days off; ATR, ataxia telangiectasia and Rad3-related kinase; cont, continuous; ER, estrogen receptor; QD, once daily; Tx, treatment.

PD biomarkers of the downstream effects of ATR inhibition (γ-H2AX and p-KAP1 Ser821) (ref. ^14^) were evaluated in 33 paired pre-treatment and on-treatment fresh biopsies collected from patients treated at doses >100 mg. Consistent with the mechanism of action of camonsertib, and confirming biologic activity at these dose levels, statistically significant increases in both γ-H2AX (*P* = 0.003, paired Wilcoxon test) and p-KAP1 (*P* < 0.001, paired Wilcoxon test) were observed (Extended Data Fig. 2).

**Fig. 2:**
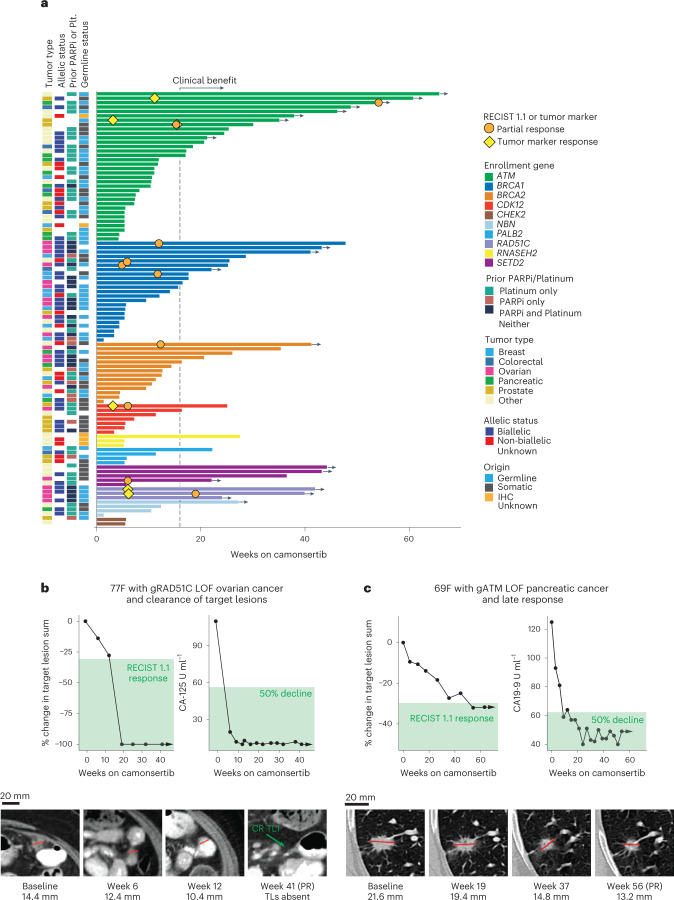
Clinical outcomes in TRESR. **a**, Duration of treatment by genotype. Clinical benefit is defined as a treatment duration of at least 16 weeks (without evidence of progression) and/or a RECIST 1.1 or tumor marker response. The gray dotted line indicates 16 weeks. **b**, Case report for a patient (*n* = 1) with g*RAD51C* LOF ovarian cancer who had complete disappearance of the TLs. **c**, Case report for a patient (*n* = 1) with a g*ATM* LOF pancreatic cancer who had a late response to camonsertib. 69F, 69-year-old female; 77F, 77-year-old female; g, genomic; Plt., platinum.

Across all tumor and molecular subtypes, 113 of 120 patients had ≥1 post-baseline tumor assessment and were evaluable for response. Of these, 12% (13/113) had a protocol-defined tumor response and the clinical benefit rate (CBR) was 42% (47/113). Of the 99 patients who received biologically effective doses of >100 mg d^−1^ of camonsertib, tumor response rate was 13% (13/99), and CBR was 43% (43/99). Median progression-free survival (mPFS) was 15 weeks (Extended Data Table 2). Responses included 10 by Response Evaluation Criteria in Solid Tumors (RECIST) 1.1 (eight confirmed partial responses (cPRs): three ovarian, two CRPC, one melanoma, one pancreatic and one head and neck squamous cell cancer (HNSCC); and two unconfirmed partial responses (uPRs): one ovarian and one breast) as well as three tumor marker responses per Prostate Cancer Clinical Trials Working Group 3 (PCWG3) or Gynecological Cancer InterGroup (GCIG) criteria (two prostate and one ovarian, respectively) (Table 3). Biomarker subgroups with responses included *ATM* (*n* = 4), *BRCA1* (*n* = 4), *RAD51C* (*n* = 2), *BRCA2* (*n* = 1), *CDK12* (*n* = 1) and *SETD2* (*n* = 1) (Fig. 2a, Table 3 and Supplementary Table 2). Additional biomarker groups with clinical benefit, but without response, included *ATM* (*n* = 10), *BRCA1* (*n* = 7), *BRCA2* (*n* = 4), *SETD2* (*n* = 3), *CDK12* (*n* = 1), *NBN* (*n* = 1), *PALB2* (*n* = 1), *RAD51C* (*n* = 1) and *RNASEH2* (*n* = 1). At the time of data cutoff, 19 patients were still receiving treatment (overall treatment duration between 5 months and 15+ months). Additionally, one patient with *ATM* LOF went on to have a RECIST partial response (PR) after data cutoff. No patients who received camonsertib at doses considered subtherapeutic had a tumor response.

**Table 3:** Responses to camonsertib monotherapy in the TRESR trial

Tumor type	Enrollment gene	Allelic status	Other features	Prior PARP inhibitor	Prior platinum	Lines of prior therapy	Time on therapy (weeks)	Response	Best % change in TL from baseline
Ovarian	g*BRCA1*	Biallelic	*BRCA1* reversion	Y	Y	6	48	RECIST cPR	−49.3
	g*BRCA1*	Biallelic		Y	Y	5	25	RECIST uPR^a^	−38.3
	g*RAD51C*	Biallelic		Y	Y	3	40+	RECIST cPR^b^	−100
	g*RAD51C*	Biallelic		Y^c^	Y	5	42+	CA-125	−12.5
	s*SETD2*	Unknown		N	Y	4	22+	RECIST cPR	−70
CRPC	s*ATM*	Unknown		N	N	2	30	RECIST cPR	−33.7
	s*ATM*	Biallelic		N	N	7	61+	PSA	−29.8
	g*ATM*	Unknown		N	N	3	35+	PSA^d^	NA
	s*CDK12*	Biallelic		N	Y	6	25	RECIST cPR	−31.9
Breast	s*BRCA*1	Biallelic		N	N	7	18	RECIST uPR	−30.4
Melanoma	s*BRCA2*	Monoallelic	TMB-H; Sig 7 (UV light)	Y	N	5	41+	RECIST cPR	−69.9
HNSCC	s*BRCA1*	Monoallelic	TMB-H; Sig 2 + 13 (APOBEC)	N	Y	1	26	RECIST cPR	−36.7
Pancreatic	g*ATM*	Unknown		N	Y	2	54+	RECIST cPR	−32.1
NSCLC	g*ATM*	Biallelic		N	Y	3	37+^e^	RECIST cPR^e^	−31.4

^a^PR unconfirmed due to progression of brain lesions though sustained reduction in TLs and 5/7 NTLs disappeared. ^b^Patient had complete response of TLs (NTL still present). ^c^Two prior PARP inhibitors. ^d^Non-measurable disease; >90% PSA decrease. ^e^uPR occurred at 37 weeks of treatment on 7 July 2022, after the 22 March 2022 data cut. ‘+’ indicates treatment ongoing at time of the 22 March 2022 data cut.

g, germline; N, no; NA, not available; s, somatic; Sig, signature; Y, yes.

**Extended Data Fig. 3:**
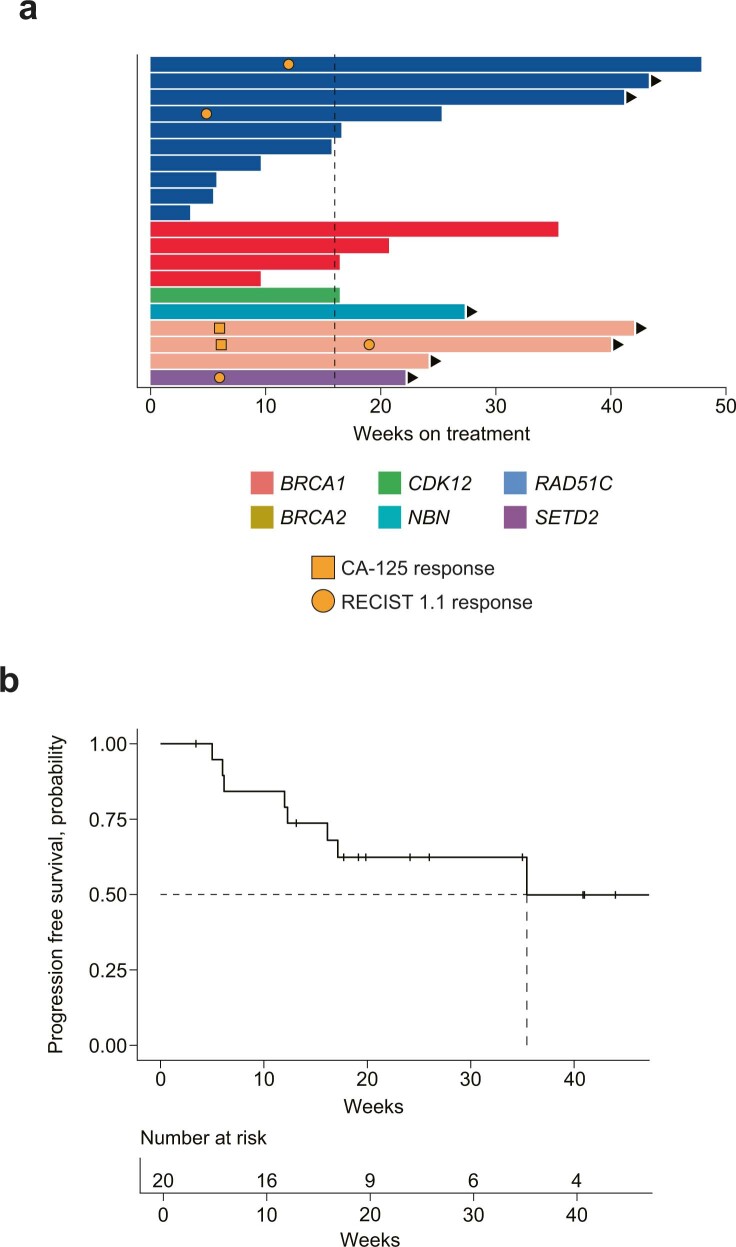
Clinical outcomes of ovarian cancer patients treated with camonsertib. (**a**) Duration of treatment. (**b**) Kaplan–Meier estimate for progression-free survival. Median progression-free survival was 35 weeks. RECIST, Response Evaluation Criteria in Solid Tumors.

Patients with ovarian cancer (*n* = 20; 82% high-grade serous) had the highest response rate (25%), highest CBR (75%) and longest mPFS (35 weeks) versus other tumor types (Extended Data Fig. 3 and Extended Data Table 3). These patients were heavily pre-treated (median six prior lines; IQR 4–7.5); most (75%; 15/20) were platinum refractory/resistant; and 90% (18/20) had prior PARPi treatment. Responses were observed in ovarian tumors with LOF alterations in *gBRCA1* (*n* = 2), *gRAD51C* (*n* = 2) and *SETD2* (*n* = 1). All responders with ovarian cancer had received prior platinum therapy and PARPi therapy, except for one patient with a granulosa cell ovarian tumor with a *SETD2* LOF alteration (Table 3 and Fig. 2a). Of interest, a 77-year-old female with ovarian cancer and a germline *RAD51C* LOF alteration, who had progressed on olaparib, had an 82% decrease in cancer antigen 125 (CA-125) at 6 weeks and an overall RECIST 1.1 PR with complete target lesion (TL) resolution at 19 weeks (Fig. 2b).

Across genomic subgroups in patients treated on trial, responses were most frequent in *BRCA1* LOF tumors (17%; 4/23: ovarian (*n* = 2), breast (*n* = 1) and HNSCC (*n* = 1)). Among *ATM* LOF tumors (*n* = 34), four (12%) patients achieved a response; 3/7 patients with *ATM* LOF CRPC had RECIST 1.1 (*n* = 1) or prostate-specific antigen (PSA, per PCWG3 criteria; *n* = 2) responses and prolonged treatment duration (≥30 weeks at the time of the data cutoff; Fig. 2). Time to response for patients with *BRCA1/2* LOF tumors versus *ATM* LOF tumors differed substantively; patients with *BRCA1/2* tumors achieved RECIST 1.1 PR at 6–12 weeks of treatment, whereas patients with *ATM* tumors displayed prolonged RECIST 1.1 stable disease or achieved PRs as late as 54 weeks. For example, a 69-year-old female with advanced pancreatic cancer harboring a germline *ATM* frameshift alteration treated with two lines of prior therapy (chemotherapy and immunotherapy) had a 50% decline in cancer antigen 19-9 (CA-19-9) at week 9 and a gradual decline in TLs, eventually resulting in a RECIST 1.1 cPR at week 54 (Fig. 2c). Further illustrating the late responses in patients with *ATM* LOF tumors, after the data cutoff, a patient with advanced non-small cell lung cancer (NSCLC) and a germline *ATM* LOF tumor had a RECIST 1.1 PR after 37 weeks of treatment (Table 3).

### Exploratory endpoints

**Fig. 3:**
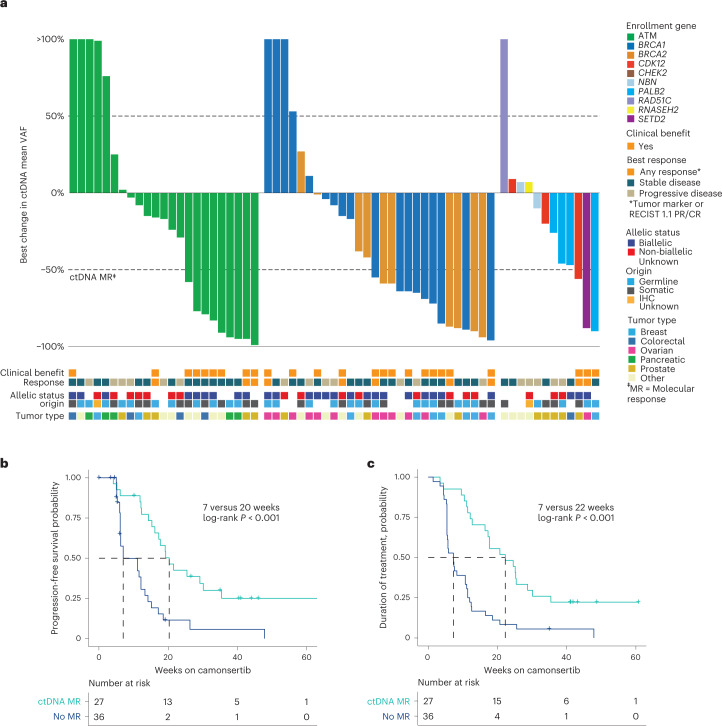
ctDNA MRs in TRESR. **a**, Best ctDNA response by enrollment gene. MR is defined as a 50% decrease from baseline in the mVAF. Kaplan–Meier-estimated PFS (**b**) and DOT by MR (**c**). log-rank *P* = 0.00015 for PFS and *P* = 0.000027 for DOT in patients with MR versus no MR. CR, complete response.

To evaluate ctDNA dynamics as a measure of camonsertib anti-tumor activity, ctDNA samples collected at baseline (88%; 106/120 patients) and longitudinally (83%; 100/120 patients) were subjected to targeted sequencing using a commercially available 105-gene liquid biopsy test. In the efficacy-evaluable population, 64% (63/99) had sufficient ctDNA levels for analysis, both at baseline and on treatment (Methods). Molecular responses (MRs), defined as a 50% decline in the mean variant allele frequency (mVAF) of somatic variants, were detected in 43% (27/63) of evaluable patients (Fig. 3a) and occurred early in treatment (median of 3.3 weeks). Across tumor types, 54% (7/13) of patients with ovarian cancer, 31% (4/13) of patients with CRPC and 70% (7/10) of patients with breast cancer (Fig. 3a and Extended Data Table 3) had MRs. Across biomarker subgroups, 39% (9/23) of *ATM*, 50% (9/18) of *BRCA1*, 60% (6/10) of *BRCA2* and 25% (3/12) of other enrollment genes had MRs, including one patient each with tumors harboring *PALB2*, *CDK12* and *RAD51C* (Fig. 3a, Supplementary Table 2 and Extended Data Table 4). The rate of MR was not significantly different across biomarker subgroups when stratified by somatic (13/28; 46%) or germline (11/22; 48%) origin (*P* > 0.99).

Among patients achieving a clinical response (tumor marker and/or RECIST 1.1) with alterations monitored longitudinally, 70% (7/10) achieved MRs and 90% (9/10) had decreases in ctDNA. Furthermore, 80% (12/15) of patients with a best clinical response of stable disease and clinical benefit had an MR. In contrast, only 25% (5/20) of patients with a best clinical response of stable disease and no clinical benefit, and 17% (3/18) of patients with a best clinical response of progressive disease, achieved MR (Fig. 3a). Patients with clinical benefit had significantly higher MR rates (76%; 19/25) than patients without (21%; 8/38) (*P* < 0.001) (Fig. 3a). We found that the enrichment for MR in patients with clinical benefit was significant within the *ATM* subgroup (clinical benefit, 83% (10/12); non-clinical benefit, 9% (2/23); *P* < 0.001) but not in the *BRCA1/2* subgroup (clinical benefit, 69% (9/13); non-clinical benefit, 40% (6/15); *P* = 0.15). Furthermore, patients with MR had significantly longer mPFS (MR, 20 weeks; no MR, 7 weeks; *P* < 0.001) and median duration of treatment (mDOT) (MR, 22 weeks; no MR, 7 weeks; *P* < 0.001) than those without (Fig. 3b,c). The lack of clinical benefit in eight patients with MR could result from early dose interruptions or reductions (3/8) or discontinuation due to PD as a result of new lesions in the setting of an overall reduced tumor burden (2/8). Patients with discordant ctDNA MR and clinical outcomes (14/63) are listed in Supplementary Table 3.

To assess the relationship between biallelic loss and clinical and molecular responses, we assessed the allelic status of the enrollment gene, which was determined in 70% (69/99) of the efficacy-evaluable group; 70% (48/69) were biallelic and 30% (21/69) were non-biallelic (Fig. 2a). Among clinical responders with known allelic status, 78% (7/9) had biallelic LOF, including two patients with germline *BRCA1*-altered ovarian cancer previously treated with PARPi and platinum therapy (Table 3). One of these patients also had a *BRCA1* reversion alteration (p.E143* > p.E143D). Other clinical responders whose tumors had biallelic LOF included two patients with CRPC (somatic *ATM* and *CDK12* alterations) and one with germline *BRCA1*-altered breast cancer. Two patients with monoallelic somatic gene loss also had responses, one with a *BRCA1* alteration (HNSCC) and one with a *BRCA2* alteration (melanoma). Both had high tumor mutational burden (TMB-H) with Catalogue of Somatic Mutations in Cancer (COSMIC) mutational signatures associated with apolipoprotein B mRNA-editing enzyme catalytic polypeptide (APOBEC) and ultraviolet (UV) light, respectively (Supplementary Fig. 4).

**Extended Data Fig. 4:**
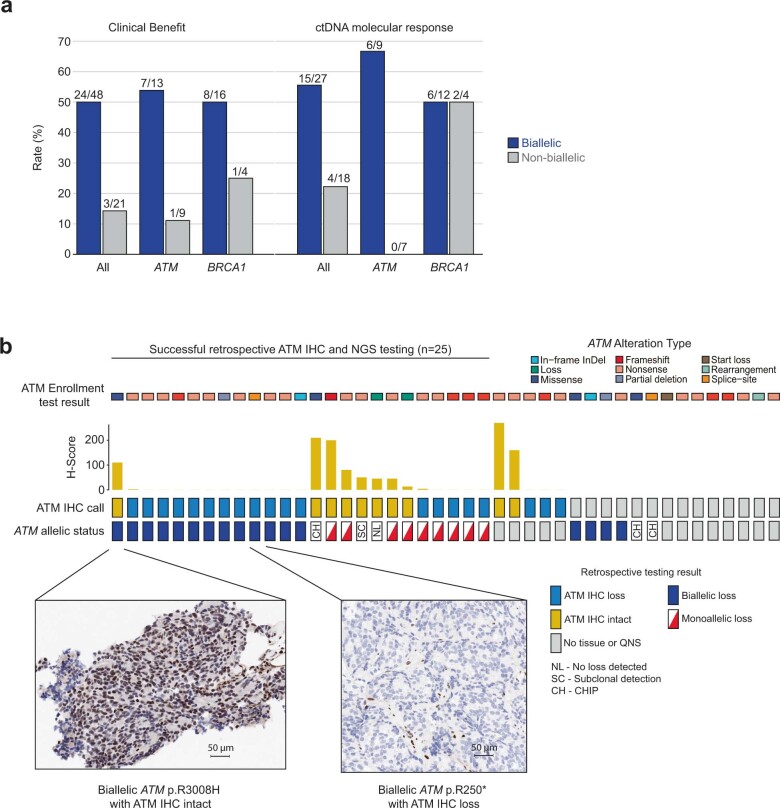
Analysis of allelic status of the enrollment gene. (**a**) Clinical benefit and molecular response rate of tumors stratified by biallelic versus non-biallelic status. (**b**) Comparison of the enrollment NGS test result for ATM with retrospective ATM IHC and allele-specific copy-number result. Micrographs show images of CRPC tissue from two of 30 patients subjected to IHC analysis with an anti-ATM antibody, illustrating cancers with intact ATM expression (ATM IHC intact) and loss of ATM expression (ATM IHC loss), respectively. CH, CHIP; CHIP, clonal hematopoiesis of indeterminate potential; CRPC, castration-resistant prostate cancer; ctDNA, circulating tumor DNA; IHC, immunohistochemistry; InDel, insertion or deletion; NGS, next-generation sequencing; NL, no loss detected; QNS, quantity not sufficient; SC, subclonal detection.

**Extended Data Fig. 5:**
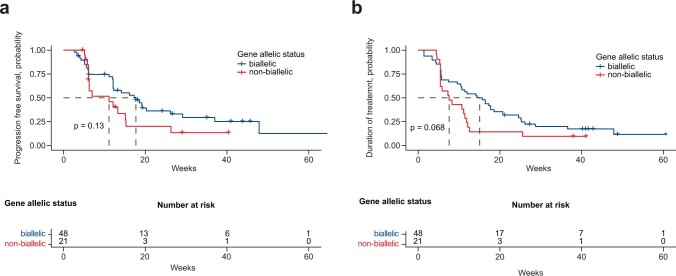
Kaplan–Meier estimate of (**a**) progression-free survival and (**b**) duration of treatment by gene allelic status.

The clinical response rate in patients whose tumors had biallelic LOF was 15% (7/48) versus 10% (2/21) in patients whose tumors had non-biallelic LOF (*P* = 0.71). Notably, a higher CBR was observed in the biallelic (50%; 24/48) versus the non-biallelic (14%; 3/21) subgroup (*P* = 0.007). Within the most common biomarker subgroups, *ATM* and *BRCA1*, patients with biallelic LOF had numerically higher CBR (*ATM*, 54% (7/13); *BRCA1*, 50% (8/16)) than patients without (*ATM*, 11% (1/9); *BRCA1*, 25% (1/4)) (Extended Data Fig. 4a). Similarly, the biallelic subgroup had numerically longer mPFS and mDOT (mPFS, 18 weeks; mDOT, 15 weeks) versus the non-biallelic subgroup (mPFS, 11 weeks; mDOT, 8 weeks) (*P* = 0.13 and *P* = 0.07, respectively) (Extended Data Fig. 5a,b). We also observed a higher MR rate for the biallelic (56%; 15/27) versus the non-biallelic (22%; 4/18) subgroup (*P* = 0.035) (Extended Data Fig. 4a).

Given that biallelic LOF correlated with higher CBR in the *ATM* biomarker subgroup (Extended Data Fig. 4a), we also evaluated the relationship between biallelic *ATM* LOF and ATM protein expression. Among the 30 patients with tumors evaluated for ATM immunohistochemical (IHC) analysis, two-thirds of samples (20/30) displayed loss of protein expression, whereas 33% (10/30) had varying levels of tumor cell ATM protein expression (median H-score 95; IQR 46–190) (Extended Data Fig. 4b). Among the 25 patients with ATM IHC results and a definitive *ATM* allelic status, those with biallelic *ATM* LOF were significantly less likely to be positive for ATM protein (8%; 1/13) than those with non-biallelic *ATM* alterations (58%; 7/12) (*P* = 0.01) (Extended Data Fig. 4b). Notably, a patient with advanced CRPC whose tumor had biallelic *ATM* LOF and was positive for ATM protein had a pathogenic missense mutation in the phosphatidylinositol 3-kinase (PI3K) regulatory domain of *ATM* (p.R3008H), which is not expected to result in loss of ATM expression (Extended Data Fig. 4b). At the data cut, this patient remains on camonsertib treatment for 61 weeks with RECIST 1.1 stable disease with tumor regression (29% reduction in RECIST 1.1 tumor measurements at last scan).

In the process of defining MRs based on ctDNA sequencing, we sequenced peripheral blood mononuclear cells (PBMCs) and identified variants derived from clonal hematopoiesis of indeterminate potential (CHIP) or the germline. Of 77 patients with both ctDNA and PBMC sequencing data, 29 had at least one variant (median one variant; IQR 1–2 variants) detected in ctDNA determined to be derived from CHIP, most commonly in *TP53* and *ATM* (Supplementary Fig. 5). Interestingly, for three patients enrolled based on *ATM* alterations (two identified by ctDNA analysis, one by tumor NGS), we determined that their enrollment alterations were derived from CHIP rather than their tumor (Supplementary Table 4). Notably, none of these patients experienced clinical benefit (Supplementary Table 4). Finally, we noted that CHIP-derived alterations in *ATM* were more common among patients with germline *ATM* alterations (57%; 8/14) than among patients with any other enrollment alterations (10%; 6/63) (*P* = 0.002) (Supplementary Fig. 5). These findings underscore the complexity and challenges of accurate molecular diagnosis of *ATM* LOF. We propose recommendations for diagnosing *ATM* alterations in Supplementary Fig. 6.

**Extended Data Fig. 6:**
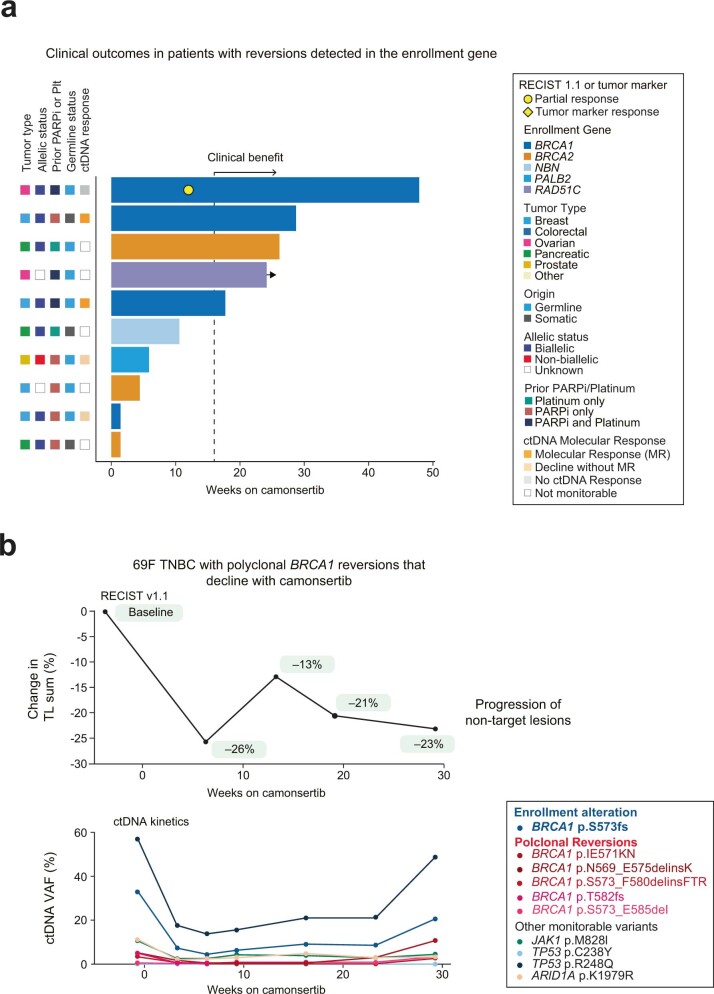
Clinical outcomes in patients harboring reversions of their tumor alterations at study enrollment. (**a**) Duration of treatment in the 10 patients in which reversion alterations were identified either by liquid biopsy or tumor sequencing. (**b**) Case report of a 69-year-old female with triple-negative breast cancer harboring polyclonal *BRCA1* reversion alterations in ctDNA. *BRCA1* reversion alterations decline with camonsertib treatment and rise prior to progression of non-target lesions. 69F, 69-year-old female; ctDNA, circulating tumor DNA; MR, molecular response; NTL, non-target lesion; PARPi, poly adenosine diphosphate-ribose polymerase inhibitor; Plt, platinum; RECIST, Response Evaluation Criteria in Solid Tumors; TL, target lesion; TNBC, triple-negative breast cancer; VAF, variant allele frequency.

In cancers with biallelic LOF alterations affecting DDR-related and HRR-related genes, resistance to cytotoxic therapies and DNA-repair-targeting agents may be mediated by somatic reversion alterations or intragenic deletions that restore the open reading frame of the gene initially affected by the LOF mutation^21^. Reversion mutations were retrospectively detected in 10 patients with primary trial enrollment alterations in *BRCA1* (*n* = 4), *BRCA2* (*n* = 3), *NBN* (*n* = 1), *PALB2* (*n* = 1) and *RAD51C* (*n* = 1), all of whom received prior PARPi (*n* = 5), platinum therapy (*n* = 2) or both (*n* = 3). Reversion alterations were detected by tissue NGS (*n* = 6), ctDNA (*n* = 2) or both (*n* = 2) (Supplementary Table 5). All 10 patients were treated with camonsertib at ≥120 mg QD (3/4 schedule); five achieved clinical benefit and one with ovarian cancer harboring a *BRCA1* reversion had a RECIST 1.1 cPR (Extended Data Fig. 6a). In a patient with a somatic *BRCA1* triple-negative breast cancer treated with two prior regimens of olaparib (monotherapy and in combination with the WEE1 inhibitor adavosertib), a *BRCA1* alteration and multiple polyclonal reversions in *BRCA1* were detected at baseline, consistent with the existence of independent clones driving resistance to previous DDR-directed therapy^22^. Upon camonsertib treatment, all variants including the reversions declined in blood and then rebounded before progression of non-target lesions (NTLs) at 29 weeks (Extended Data Fig. 6b). In another patient with germline *BRCA1*-altered breast cancer and multiple reversion mutations, decreases in variant allele frequencies (VAFs) of the individual *BRCA1* reversions were observed. Despite a 26% decrease in TLs at the first scan, this patient discontinued treatment due to clinical progression after a 3-week dose hold due to an unrelated adverse event (Supplementary Fig. 7).

## Discussion

Here we present results from the phase 1 TRESR study, which represents, to our knowledge, the most comprehensively analyzed, prospectively selected cohort of tumors treated with ATRi monotherapy to date. The safety and tolerability profile of camonsertib was consistent with a highly selective and potent ATRi, and preliminary anti-tumor activity was demonstrated in heavily pre-treated tumors across a range of histologic types and enrollment gene alterations.

Since the discovery of synthetic lethality and the approval of PARPis in multiple *BRCA1/2*-deficient homologous recombination deficiency (HRD)-associated tumor contexts, research has been ongoing to extend this therapeutic strategy to other DDR-targeting agents and genetic backgrounds, in both PARPi-naive and PARPi-resistant settings^7,23^. Previous trials have largely focused on combinations of ATRi with chemotherapy or PARPi. In the few trials of ATRi monotherapy, response rates were approximately 6–7% in unselected patients^23,24^. A study of BAY1895344 in patients selected for HRD alterations reported 4/11 patients with a response^7^, but this was not confirmed in a larger series (5/138) (ref. ^25^). In TRESR, although response rates across the entire efficacy cohort, comprising different tumor and molecular subtypes, were modest, patients with advanced, molecularly selected ovarian cancer had a 25% response rate and a 35-week mPFS, despite prior progression on multiple lines of therapy (including platinum chemotherapy and PARPi). We hypothesize that ovarian cancers may be vulnerable to ATRi because of their intrinsically high replication stress, loss of tumor suppressors and high frequency of biallelic DDR gene loss^26–30^.

Within the ovarian cancer subset, camonsertib anti-tumor activity was observed in patients with *BRCA1*-altered tumors previously treated with PARPi or platinum therapy, most notably in a post-PARPi-treated patient with a germline *BRCA1* alteration, despite the presence of reversion mutations. Although reversions (for example, *BRCA1*, *BRCA2* and *PALB2*) are sufficient to drive PARPi resistance^22,31,32^, these acquired alterations may not entirely restore HRR function. Thus, it is plausible that PARPi-resistant cancers may be sensitive to ATRi, addressing an unmet medical need. Although the small, heterogeneous population of patients with *BRCA1*-altered cancers did not allow formal statistical analysis, CBR in this population was approximately 48%. These data warrant exploration in future clinical trials to confirm the role of *BRCA1* status as a biomarker of ATRi sensitivity.

Time to response was shorter in patients with *BRCA1*-altered tumors than in patients with *ATM*-altered tumors. The reason for the late responses in *ATM*-altered tumors is not well understood. Given that some data suggest that *BRCA1-*altered tumors display a high proliferative index^33–39^, and that ATRis are thought to kill cells predominantly in the G2/S phase of the cell cycle^40^, we hypothesize that the faster time to response for *BRCA1*-altered tumors may be related to their relatively higher proliferation rate.

Notably, TRESR also included patients harboring DDR-altered genes for which no targeted standard-of-care therapy exists, such as *SETD2*. The response in a patient with *SETD2*-altered ovarian cancer may reflect the relevant role of *SETD2* in suppressing DNA damage and replication stress through regulation of nucleosome stability^41^ and by maintaining cellular dNTP levels during DNA replication^42^. These results demonstrate that DDR-targeting agents may be clinically active in other patient populations, including those with other genomic (*ARID1A*, *CCNE1* and *MYC*)^12,43,44^ or phenotypic (replication stress)^45^ markers, warranting future clinical studies in these contexts.

These observations suggest that a better understanding of the factors that predict clinical responses to ATRi is needed to help guide the design of future trials developing ATRis^46,47^. Previous studies from our group and others have shown that biallelic (but not monoallelic) LOF of *BRCA1/2* is associated with features of HRD and dysfunctional DDR pathways vulnerable to synthetic lethal therapeutic strategies^48–54^. Indeed, patients whose tumors had biallelic LOF alterations at enrollment constituted most characterized responders, with significantly higher rates of MR and clinical benefit, consistent with the hypothesis that camonsertib is more active in tumors with biallelic LOF in predicted ATRi-sensitizing genes.

Interestingly, responses were observed in two patients with monoallelic *BRCA1/2-*altered tumors (HNSCC and melanoma) that are not part of the canonical spectrum of *BRCA1*/2-associated cancers (hereditary breast, pancreatic, prostate and ovarian cancers). Both tumors were TMB-H and had COSMIC mutational signatures not indicative of deficiencies in the HRR or DDR pathways traditionally observed in *BRCA*-associated tumors. A recent report demonstrated that patients with TMB-H NSCLC treated with a combination of the ATRi berzosertib and gemcitabine had higher response rates versus patients without elevated TMB^55^. Although preliminary, these data suggest a possible role for ATRi in patients with TMB-H tumors.

To better understand the optimal method for identifying *ATM* LOF, we assessed the concordance of *ATM* allelic status and ATM protein loss by IHC. Although detection of biallelic *ATM* loss by tumor sequencing was strongly predictive of ATM protein loss by IHC, pathogenic mutations affecting *ATM* that do not lead to a premature stop codon and nonsense-mediated decay may cause false-positive IHC results, even in the presence of bona fide biallelic LOF. Notably, a response was observed in a patient with CRPC harboring biallelic *R3008H* LOF alterations while retaining ATM protein expression. These results underscore the importance of determining the allelic status of *ATM* LOF alterations to optimize patient selection for treatment with ATRi.

The relatively high prevalence of *ATM* CHIP mutations presents another challenge to accurately measuring *ATM* LOF in ctDNA^56^. Deep targeted sequencing of PBMCs performed in this study allowed for refinement of tumor-uninformed ctDNA analysis by filtering variants derived from germline or CHIP and focusing on somatic variants that were most likely to be tumor derived. Interestingly, three patients were found to have been enrolled with *ATM* alterations derived from PBMCs. The contamination of CHIP-derived variants in solid and liquid tumor NGS is widespread, as the implementation of matched PBMC sequencing is not universally adopted in clinical genomics laboratories, especially for ctDNA analysis^22,53,54^. Our findings demonstrate that CHIP variants affecting *ATM*, as well as other tumor suppressor genes, may confound the interpretation of plasma DNA-only sequencing (that is, without concurrent NGS on matched PBMC DNA). This highlights the importance of CHIP filtering when enrolling patients on trials using ctDNA or tumor-only NGS and when interpreting MRs from tumor-uninformed ctDNA analysis.

We also evaluated whether ctDNA changes can serve as a surrogate marker of therapeutic response, as previously reported^51,52^. Most patients who achieved stable disease as best clinical response by RECIST 1.1 had MRs, supporting the hypothesis that anti-tumor activity, measured by ctDNA changes, contributed to the prolonged disease stabilization. These data and the observed correlation of MR with outcomes in this and other trials^51,52^ strongly support the anti-tumor activity of camonsertib in these patients, despite limited observable tumor shrinkage. Although MR and clinical benefit were concordant in most patients, a subset of patients had discordant results. These discordant cases could generally be categorized by (1) patients with low baseline mVAFs (<1%), in which results may be confounded by stochastic variation of the commercial ctDNA panels used; (2) patients with aggressive and/or heterogenous cancers who had an initial MR that soon after rebounded concurrently with disease progression; and (3) patients with an MR who had subsequent dose interruptions and/or unrelated adverse events that limited drug exposure, thus confounding the comparison.

This study has several limitations. As an early-phase trial, TRESR is a non-comparative study in a heavily pre-treated patient population with genomically complex, treatment-resistant, heterogeneous tumors. Enrolling patients with tumors harboring pathogenic mutation genes other than *ATM, BRCA1* and *BRCA2* was challenging owing to the low prevalence of pathogenic alterations affecting these genes in patients with metastatic cancer (<1%). Although only patients with prospectively identified alterations were enrolled, this phase 1 trial was open to patients with any advanced solid tumor with no restriction on prior lines of therapy. Therefore, within each genotype, there was a variety of tumor types, histology, allelic status, germline status, prior therapies and other characteristics that may explain the heterogeneity in responses observed within each class of alterations in this trial. Context dependency between different molecular alterations and tumor types may also impact sensitivity to camonsertib. Despite these challenges, RECIST 1.1 responses were observed in patients with rare genotypes, including *SETD2*, *CDK12* and *RAD51C*, and clinical benefit was observed in patients with tumors harboring *CDK12*, *NBN*, *PALB2*, *RAD51C, RNASEH2* and *SETD2* alterations. Future studies are required to investigate camonsertib in tumors harboring alterations across the SNIPRx LOF genes, particularly the rarer genotypes not enrolled in this study (that is, *REV3L*, *RAD51D*, *RAD50*, *RAD17*, *MRE11*, *FZR1*, *CHTF8* and *ATRIP*).

The results of the TRESR trial highlight the utility of pre-clinical results from chemogenomic CRISPR–Cas9 screens and small-scale validation experiments^9–19^ for informing patient enrollment and stratification in trials based on principles of synthetic lethality, and the testable hypotheses generated have clear implications for the development of ATRis and other DDR-targeted agents. Multiple clinical trials of camonsertib alone and in combination with other therapies are ongoing, to further refine the subgroups of patients where camonsertib is most active (NCT04855656, NCT04972110 and NCT05405309). The collective body of evidence supports the further development of camonsertib, particularly in, but not limited to, tumors such as ovarian cancer.

## Methods

### Study design and treatment

TRESR (NCT04497116) is a modular, phase 1/2a, first-in-human, multicenter, open-label, non-randomized, dose-escalation, dose-expansion study of camonsertib, administered orally as a single agent or in combination with talazoparib or gemcitabine in patients with advanced solid tumors. The results reported here focus on module 1, which includes the dose escalation of camonsertib monotherapy, and is further divided into three submodules: module 1a (M1a), 5/2 dosing schedule; module 1b (M1b), 3/4 dosing schedule; and module 1c (M1c), food effect evaluation. For the 12 patients enrolled in M1c, camonsertib was administered with a high-fat, high-calorie meal on day −3 and in the fasted state on day 1, to evaluate the effect of food on the PK of camonsertib. After the food effect portion of the study, patients continued camonsertib monotherapy on either the 5/2 or 3/4 schedule and were analyzed for safety and efficacy along with the patients in M1a and M1b. The study was conducted in accordance with the Declaration of Helsinki and Council for International Organizations of Medical Sciences International Ethical Guidelines, applicable International Conference on Harmonization Good Clinical Practice Guidelines and applicable laws and regulations. All patients provided written informed consent to adhere to the clinical protocol and provide serial blood samples and tumor tissue. The protocol was approved by the institutional review board or ethics committee at each participating institution.

Patients were treated with single-agent camonsertib at doses ranging from 5–160 mg QD to 40–80 mg twice daily, administered orally on a 5/2 or 3/4 weekly schedule. An intermittent schedule of 2 weeks on/1 week off was also evaluated at dose levels of 160 mg and 200 mg QD (3/4). Each cycle comprised 21 d of treatment. Module 1 dose-escalation decisions based on patients in M1a and M1b cohorts were governed by the Bayesian optimal interval (BOIN) design starting with single-patient cohorts in M1a. Evidence of pharmacologic activity—defined as grade ≥2 drug-related toxicity at any cycle, PK data demonstrating exposure levels predicted to be efficacious based on non-clinical studies or other evidence of treatment-related activity—served as the trigger for cohort expansion and opening of M1b. Escalation of M1a and M1b occurred in parallel until the maximum tolerated dose (MTD) of RP2D was determined for each schedule. Backfill cohorts were employed to (1) aid in the assessment of possible anti-tumor activity in a subset of patients with a specific gene abnormality or tumor type; (2) allow additional PK/PD evaluation; and (3) further assess drug-related toxicities. Patient safety review and dose decisions were carried out by a Safety Review Committee, comprised of the study investigators and sponsor representatives.

### Gene panel design

To select genes included on our enrollment panel, we mined internal and external CRISPR–Cas9 screen datasets^9,10,13^. First, we selected gene hits that overlapped between the published consensus set of ATRi-sensitizing alterations^9^ and our internal camonsertib screen^13^. Of these 35 genes^13^, we selected those that (1) showed LOF alterations in tumor genomic datasets (for example, The Cancer Genome Atlas and Project GENIE); (2) could be identified by targeted NGS panels (internal or commercial); and (3) led to ATRi sensitivity when inactivated with CRISPR–Cas9 or RNAi in internal or published experiments^9,10,12–14^. This resulted in a list of eight genes (*ATM*, *ATRIP*, *BRCA2*, *RAD17*, *RAD51B*, *RNASEH2A/B* and *SETD2*). We then supplemented this list with additional genes based on the same criteria but that were (1) unique to either the Hustedt et al.^9^ or Zimmermann et al.^13^ datasets or (2) present in well-described functional pathways accompanying the genes on the initial list (*BRCA1*, *PALB2*, *RAD51C/D*, *CDK12*, *FZR1*, *CHTF8*, *REV3L* and *MRE11-NBN-RAD50*).

### Patients

A data cutoff date of 22 March 2022 was used for safety and efficacy analyses. Patients in module 1 (*n* = 120 enrolled as of 1 November 2021) entered the trial across 12 sites in North America (United States and Canada) and Europe (United Kingdom and Denmark).

Full inclusion criteria included: signature of written informed consent form by the patient or legal guardian; adult aged ≥18 years at the time of consent; ECOG performance status score of 0 or 1; histologically confirmed solid tumor resistant or refractory to standard treatment and/or patients intolerant to standard therapy; measurable disease per RECIST 1.1 (allowance made upon sponsor approval for enrollment of patients without measurable disease if monitorable by tumor markers); provision of archival tumor tissue sample or fresh biopsy; ability to comply with protocol and study procedures; ability to swallow and retain oral medications; acceptable organ and hematologic function at time of screening; negative pregnancy test for women of childbearing potential at time of screening and before first dose; willingness to use highly effective contraception during the study period and 6 months after last dose; resolution of toxicities of prior therapy or surgery; completion of any radiation therapy 7 d before first dose; life expectancy ≥12 weeks after the start of treatment; (M1c only) ability to consume a high-fat meal and fast for 12 h.

All eligible patients had deleterious or likely deleterious gene alterations for at least one of the following genes: *ATM*, *ATRIP*, *BRCA1*, *BRCA2*, *CDK12*, *CHTF8*, *FZR1*, *MRE11*, *NBN*, *PALB2*, *RAD17*, RAD50, *RAD51B/C/D*, *REV3L*, *RNASEH2A*, *RNASEH2B*, *SETD2* or other genes agreed upon between the sponsor and investigator (for example, *CHEK2*). Upon pre-screening consent, available NGS results (germline, tumor or ctDNA) from Clinical Laboratory Improvement Amendments/College of Pathology, International Organization for Standardization or equivalent certified laboratories were centrally confirmed and annotated by the Precision Oncology Decision Support group^57^ at The University of Texas MD Anderson Cancer Center. Tumors with RNASEH2B protein loss were screened and identified with an RNASEH2B IHC (RbMab) clinical trial assay (NeoGenomics). RNASEH2B loss was defined as 0–10% positive tumor cells.

Patients were not eligible to participate if they met any of the following exclusion criteria: treatment with chemotherapy, small molecule or biologic anti-cancer therapy within 14 d before first dose of study drug; prior therapy with an ATR or DNA-PK inhibitor; history of or current condition, therapy or laboratory anomaly that could compromise patient safety, confound study results or interfere with study participation; known hypersensitivity to any ingredients of camonsertib; uncontrolled, symptomatic brain metastases; uncontrolled hypertension; active, uncontrolled bacterial, fungal or viral infection; moderate or severe hepatic impairment; clinically significant history of abnormal electrocardiogram, history or risk of ventricular dysrhythmias, Fridericia formula for corrected QT interval (QTcF) >470 ms or treatment with medications known to prolong QT interval; history of myelodysplastic syndrome or acute myeloid leukemia; inability to comply with protocol and follow-up procedures; treatment with strong CYP3A inhibitors or inducers, P-gp inhibitors or BCRP inhibitors within 14 d of first dose; and pregnancy or breastfeeding.

### Study endpoints

Tolerability and safety of camonsertib was evaluated by assessment of AEs, TEAEs, serious adverse events (SAEs), DLTs, concomitant medications and procedures, physical examinations, vital sign measurements, clinical safety laboratory evaluations (hematology, chemistry and urinalysis), ECOG performance status scores and electrocardiograms.

The exploratory efficacy endpoint was assessment of anti-tumor activity by overall response rate, duration of treatment (DOT), CBR, PFS and overall survival. Overall response rate was defined as the proportion of patients with best response of complete response or PR according to RECIST 1.1, confirmed CA-125 response based on GCIG criteria or PSA response based on PCWG3. CBR was defined as the proportion of patients with a response by RECIST 1.1 or confirmed CA-125 by GCIG criteria or PSA response based on PCWG3 or a treatment duration of at least 16 weeks without prior evidence of progression (modification from the original protocol definition). Tumor responses were assessed according to RECIST 1.1 every 6 weeks for the first three assessments and thereafter every 9 weeks. Serum tumor biomarkers were assessed once per cycle or as per standard of care. For a tumor marker responder, there must also have been no evidence of radiologic or clinical progression before or within 4 weeks of the initial response. mPFS was calculated using the Kaplan–Meier method, where a disease progression per RECIST or death was treated as an event. Those patients without events were censored at their last tumor assessment date before the cutoff date. Similarly, to calculate mDOT, patients who discontinued treatment were treated as events, and those without events were censored at the data cutoff date.

### PK

The plasma levels from cycle 1, day 1 of camonsertib were quantified using a validated liquid chromatography tandem–mass spectrometry (LC–MS/MS) method. PK parameters for RP-3500 were calculated using non-compartmental analysis using Phoenix version 8.3.3.33 (Certara): AUC from time 0 to last quantifiable concentration (AUC_0–last_); AUC from time 0 to infinity (AUC_0–inf_); AUC from 0 h to 12 h after dose (AUC_0–12_); maximum observed plasma concentration (C_max_); T_max_; and terminal elimination half-life (t_½_) were calculated. All PK parameters were calculated using actual sampling times. Mean plasma concentration time profiles at given dose levels and regimens were plotted on semi-logarithmic plots using nominal times.

### Statistical analysis

The planned maximum total number of patients exposed in module 1 was 140. The actual number of patients was determined by the number of dose escalations and patients enrolled in any of the backfill cohorts (up to eight each). This number was deemed sufficient to enable a better understanding of drug-related toxicity, at dose levels where the initial treatment effect was observed. Dose-escalation decisions were governed by the BOIN design^58^ and confirmed at the Safety Review Committee meetings at the end of the DLT observation period.

All safety and efficacy endpoints were summarized with descriptive statistics. Safety data were summarized using the safety population, which consisted of all patients who received at least one dose of RP-3500. The DLT rate was based on the DLT-evaluable population, which included those patients in dose-escalation cohorts (M1a and M1b only) receiving ≥80% of planned doses of camonsertib, who completed all required safety evaluations and were observed through the end of cycle 1 or patients who experienced a DLT. Due to the different number of dose levels expected and the exploratory nature of the phase 1 study, anti-tumor activity parameters were primarily based on patients who received ≥1 dose of RP-3500 and had ≥1 post-baseline tumor assessment by RECIST 1.1 and/or GCIG CA-125 or PCWG3 PSA criteria, with an initial dose level of >100 mg d^−1^ (dose level predicted to achieve efficacious exposure), as defined in the statistical analysis plan. Additional subgroups based on tumor types or genotypes of interest were based on this efficacy analysis set. There is no evidence to suggest that the efficacy of RP-3500 will be affected by sex, race or gender. Patients were, therefore, enrolled in the study and endpoints assessed regardless of sex, gender and race. No formal statistical tests were performed in this study. However, nominal *P* values (two-sided) were provided for the hypothesis-generating purpose of selected exploratory endpoints, without adjusting for multiplicity. A log-rank test was used in between-group comparisons for the time-to-event endpoints (PFS and DOT), and Fisher’s exact test was used in between-group comparisons for the binary endpoints (CBR, MR, etc.).

### Tissue sequencing with SNiPDx

#### Library preparation and sequencing

DNA (minimum of 30 ng) was extracted from 10 5-µm formalin-fixed, paraffin-embedded slides or pelleted PBMCs (minimum 120 ng). DNA samples were analyzed on a custom anchored multiplex (AMP) polymerase chain reaction (PCR) panel comprising 26 genes referred to as SNiPDx (Repare Therapeutics)^20^. Libraries were quantitated using quantitative PCR (Kapa Biosystems) per the manufacturer’s protocol. Amplicon sequencing was performed on the NovaSeq platform (Illumina) according to the manufacturer’s standard protocol. Paired-end sequence data were processed using methods developed for AMP to align error-corrected reads^31^. AMP libraries were processed using the VariantPlex Pipeline from Archer Analysis Platform version 6.2.8.

#### Variant analysis

Variants were called using LoFreq (version 2.1.1), FreeBayes (version 0.9.9) and proprietary methods. VariantPlex pipeline parameters were adjusted to accommodate the large footprint of SNiPDx and minimize background noise within variant calls. Variant calls <300 base pairs from the nearest gene-specific primers within regions of interest in reads with a minimum base quality of 22, and with a minimum allele fraction of 0.02, were reported. Genes, transcripts and consequences of variants were accessed through Alamut Batch (Interactive BioSoftware) using database version 1.5-2020.11.25. A variant call format file for each sample was generated using VCFtools version 0.1.11.

#### Allele-specific copy number analysis

Genome-wide major and minor copy numbers were inferred by FACETS^32^. An adaptive panel of normal (PoN) selection scheme was added to the standard FACETS workflow to match quality parameters to an analyzed formalin-fixed, paraffin-embedded tumor sample. Copy number alterations and allelic imbalances in the 26 SNiPDx target genes and other genomic regions were calculated on the basis of the log_2_R (that is, the log_2_ ratio of single-nucleotide polymorphism (SNP) coverage in a tumor sample to coverage in a matched normal sample or PoN), and log_2_ odds ratio (log_2_OR, calculated from the number of reads reporting the alternative allele:number of reads reporting the reference allele), adjusted by tumor purity and ploidy^32^. Minor allele (b-allele) fraction for each SNP was defined as the ratio of reads reporting the alternative allele:total number of reads at that position. Loss of heterozygosity (LOH) was determined if the minor allele copy number was zero. Samples were manually reviewed for technical parameters of sequencing and tumor content, and LOH status per gene was curated for possible mis-segmentations using plots generated from FACETS solutions.

#### Allelic status determination

Allelic status for enrollment genes was determined by SNiPDx, WGS or local NGS (where available). Enrollment genes were considered to have biallelic LOF if one of the following criteria was met: (1) homozygous deletion; (2) compound heterozygous mutation; (3) mutation and LOH; or (4) mutation and non-overlapping loss. Enrollment genes were considered to have monoallelic loss if the following criteria were met: (1) mutation without LOH or (2) heterozygous loss, considered to have no loss if no mutation or copy number loss was detected. CHIP was determined in cases where the enrollment alteration was detected in PBMCs but determined not to be germline. Subclonal alterations were those where the enrollment alteration was detectable at lower-than-expected VAF, upon adjustment for tumor purity, ploidy and local copy number, or present only in some tumor biopsies. If central results were not available and local testing could detect any of the above events leading to biallelic loss, the gene was considered to have biallelic loss. Allelic status calls were reviewed by an external board-certified molecular pathologist.

#### CHIP determination

CHIP determination was performed on the set of patients with both ctDNA and SNiPDx PBMC results. The most prominent CHIP genes (*DNMT3A*, *ASXL1* and *TET2*) were not included on either the ctDNA or the PBMC panel so are not considered in this analysis. After excluding germline alterations, CHIP was defined as alterations where the ratio of VAFs between PBMC and ctDNA was ≥25% with sufficient read support in PBMCs (≥5 reads). Additional evidence from tumor NGS was used for enrollment gene CHIP filtering (that is, if the VAF in tissue was substantially lower than PBMC). All CHIP variants were reviewed manually.

#### Mutational signatures

Mutational signatures were decomposed using DeconstructSig^59^, which is based on a multiple linear regression model to compute an optimized combination of exposures using a predefined set of signatures, and SigProfiler, which is based on non-negative matrix factorization, extracts ex novo signatures and was developed more recently on a larger cohort of patients with cancer. The function SigProfilerSingleSample was employed to obtain the decomposition of mutational signatures per patient^60^. Mutational signature exposures obtained with each method for each sample were compared and considered robust if agreement between methods was observed.

#### ctDNA analysis

Plasma samples were collected from patients at baseline at each cycle of treatment. ctDNA analysis was performed using Tempus xF (Tempus). Germline and CHIP variants were filtered by comparison with targeted sequencing of matched PBMCs. Artifacts and additional suspected germline variants were removed by manual curation. To be considered monitorable, individual variants had to have a VAF of >0.5% at any timepoint, and patients had to have at least one variant with a VAF of >1% at any timepoint. The mVAF was calculated for each timepoint for each patient, and then the mVAF ratio (mVAFR) of each on-treatment timepoint relative to baseline was calculated. For patients with multiple on-treatment timepoints, the best mVAFR was selected. Patients with ≥50% reduction in mVAFR from baseline for at least one timepoint were considered molecular responders.

### IHC

Tumor biopsies were collected from patients at baseline and on cycle 2, day 10 between 8 h and 24 h after dosing. Distal PD markers, pKAP1 and gH2AX, were then assessed by tumor IHC centrally at HistoWiz, Inc. Although CHK1 phosphorylation (p-CHK1) is a more direct and proximal biomarker of ATR inhibition, preclinical studies have demonstrated that p-CHK1 declines rapidly after dosing^61^, suggesting that tumor biopsies would need to be conducted within 2 h. Thus, p-CHK1 was not assessed owing to limited feasibility. Unstained slides sectioned at 4 μm were labeled for gH2AX Ser139 (clone 20E3, 9718, Cell Signaling Technology, 1:1,000 dilution) and pKAP1 S824 (clone BL-246-7B5, ab243870, Abcam, 1:600 dilution) on a BOND RX autostainer (Leica Biosystems) with enzyme treatment (1:1,000) and BOND Polymer Refine Detection (Leica Biosystems) according to the manufacturer’s protocol. After staining, sections were dehydrated and film-coverslipped using a TissueTek Prisma and Coverslipper (Sakura Fintech). Retrospective ATM IHC was performed by a sponsor-approved central laboratory (anti-ATM clone Y170, ab32420, Abcam, 1:250 dilution). All slides were interpreted by a board-certified pathologist. ATM loss was defined as ≤5% of positive tumor cells.

### Participating institutes

Clinical data were collected at The University of Texas MD Anderson Cancer Center; the Sarah Cannon Research Institute UK; the Dana-Farber Cancer Institute; the Sarah Cannon Research Institute/Tennessee Oncology; the University Hospital of Copenhagen; the Princess Margaret Cancer Centre; Duke University; Rhode Island Hospital; the Northern Centre for Cancer Care; Massachusetts General Hospital Cancer Center; Memorial Sloan Kettering Cancer Center; and The Christie Foundation.

### Reporting Summary

Further information on research design is available in the Nature Portfolio Reporting Summary linked to this article.

## Online content

Any methods, additional references, Nature Portfolio reporting summaries, source data, extended data, supplementary information, acknowledgements, peer review information; details of author contributions and competing interests; and statements of data and code availability are available at 10.1038/s41591-023-02399-0.

### Supplementary information

Supplementary Information Supplementary Tables 1–5 and Supplementary Figs. 1–7

Reporting Summary

## Figures and Tables

**Extended Data Table 1:**
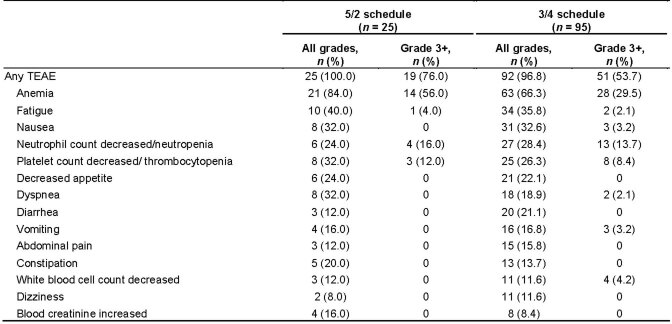
Most common TEAEs occurring in ≥10% of total treated population

3/4, 3 d on/4 d off; 5/2, 5 d on/2 d off; TEAE, treatment-emergent adverse event.

**Extended Data Table 2:**
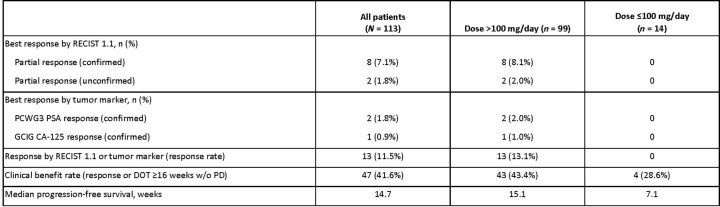
Summary of anti-tumor activity for all response-evaluable patients (≥1 post-baseline tumor assessment)

Data cutoff date was 3 March 2022. DOT, duration of treatment; GCIG, Gynecologic Cancer Intergroup; PCWG3, Prostate Cancer Working Group 3; PD, progressive disease; PSA, prostate-specific antigen; RECIST, Response Evaluation Criteria in Solid Tumors; w/o, without.

**Extended Data Table 3:**
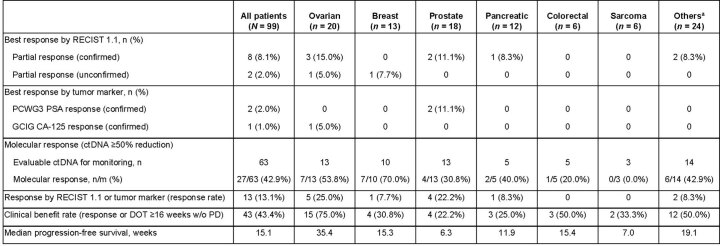
Summary of anti-tumor activity by tumor type in efficacy population with dose >100 mg d^−1^

Data cutoff date was 3 March 2022. ^a^Includes lung (*n* = 5), gastrointestinal (*n* = 5), endometrial (*n* = 3), head and neck (*n* = 2), melanoma (*n* = 2), bile duct (*n* = 1), cervical (*n* = 1), liver (*n* = 1) and four patients with other tumor types: appendiceal (*n* = 1), malignant pleural mesothelioma (*n* = 1), mesothelioma (*n* = 1) and squamous cell carcinoma—primary unknown (*n* = 1). ctDNA, circulating tumor DNA; DOT, duration of treatment; GCIG, Gynecologic Cancer Intergroup; PCWG3, Prostate Cancer Working Group 3; PD, progressive disease; PSA, prostate-specific antigen; RECIST, Response Evaluation Criteria in Solid Tumors; w/o, without.

**Extended Data Table 4:**
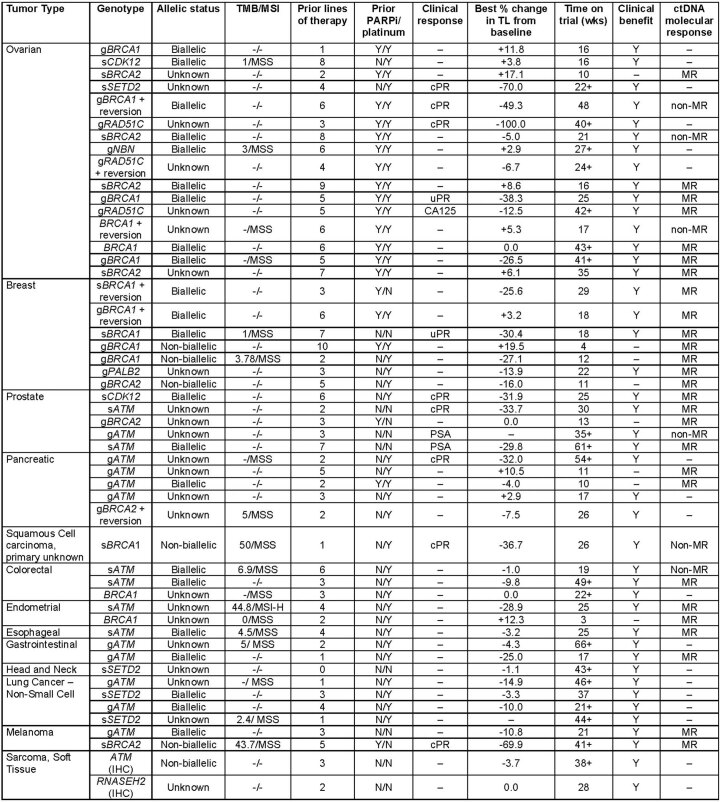
Summary of clinical and molecular characteristics of patients with clinical response, clinical benefit and MR

Data cutoff date was 22 March 2022. ‘+’ indicates that the treatment is still ongoing. Clinical benefit defined as having RECIST 1.1/tumor marker response or time on treatment ≥16 weeks without PD. g, germline; IHC, immunohistochemistry; MR, molecular response; MSI, microsatellite instability; MSI-H, microsatellite instability high; MSS, microsatellite stable; N, no; PARPi, poly adenosine diphosphate-ribose polymerase inhibitor; PSA, prostate specific antigen; s, somatic; TL, target lesion; TMB; tumor mutation burden; uPR, unconfirmed partial response; wks, weeks; Y, yes.

## Data Availability

For eligible studies, qualified researchers may request access to individual patient-level clinical data through a data request platform. At the time of writing, this request platform is Vivli (https://vivli.org/ourmember/roche/). Datasets can be requested 18 months after a clinical study report has been completed and, as appropriate, once the regulatory review of the indication or drug has completed. Access to patient-level data from this trial can be requested and will be assessed by an independent review panel, which decides whether the data will be provided, taking the risk of patient re-identification into consideration. Once approved, the data are available for up to 24 months. Anonymized records for individual patients across more than one data source external to Roche cannot, and should not, be linked owing to a potential increase in risk of patient re-identification. For up-to-date details on Roche’s Global Policy on the Sharing of Clinical Information and how to request access to related clinical study documents, see https://go.roche.com/data_sharing.

## References

[1] Hanahan D (2022). Hallmarks of cancer: new dimensions. Cancer Discov..

[2] Durocher D, Jackson SP (2001). DNA-PK, ATM and ATR as sensors of DNA damage: variations on a theme?. Curr. Opin. Cell Biol..

[3] Gaillard H, Garcia-Muse T, Aguilera A (2015). Replication stress and cancer. Nat. Rev. Cancer.

[4] O’Connor MJ (2015). Targeting the DNA damage response in cancer. Mol. Cell.

[5] Setton J (2021). Synthetic lethality in cancer therapeutics: the next generation. Cancer Discov..

[6] Pilie PG, Tang C, Mills GB, Yap TA (2019). State-of-the-art strategies for targeting the DNA damage response in cancer. Nat. Rev. Clin. Oncol..

[7] Yap TA (2021). First-in-human trial of the oral ataxia telangiectasia and RAD3-related (ATR) inhibitor BAY 1895344 in patients with advanced solid tumors. Cancer Discov..

[8] Dunlop CR (2020). Complete loss of ATM function augments replication catastrophe induced by ATR inhibition and gemcitabine in pancreatic cancer models. Br. J. Cancer.

[9] Hustedt N (2019). A consensus set of genetic vulnerabilities to ATR inhibition. Open Biol..

[10] Wang C (2019). Genome-wide CRISPR screens reveal synthetic lethality of RNASEH2 deficiency and ATR inhibition. Oncogene.

[11] Olivieri M (2020). A genetic map of the response to DNA damage in human cells. Cell.

[12] Williamson CT (2016). ATR inhibitors as a synthetic lethal therapy for tumours deficient in ARID1A. Nat. Commun..

[13] Zimmermann M (2022). Guiding ATR and PARP inhibitor combinationswith chemogenomic screens. Cell Rep..

[14] Roulston A (2022). RP-3500: a novel, potent, and selective ATR Inhibitor that is effective in preclinical models as a monotherapy and in combination with PARP inhibitors. Mol. Cancer Ther..

[15] Mohni KN, Kavanaugh GM, Cortez D (2014). ATR pathway inhibition is synthetically lethal in cancer cells with ERCC1 deficiency. Cancer Res..

[16] Fagan-Solis KD (2020). A P53-independent DNA damage response suppresses oncogenic proliferation and genome instability. Cell Rep..

[17] Katsura M (2009). The ATR-Chk1 pathway plays a role in the generation of centrosome aberrations induced by Rad51C dysfunction. Nucleic Acids Res..

[18] Saxena S, Dixit S, Somyajit K, Nagaraju G (2019). ATR signaling uncouples the role of RAD51 paralogs in homologous recombination and replication stress response. Cell Rep..

[19] Gomes LR (2019). ATR mediates cisplatin resistance in 3D-cultured breast cancer cells via translesion DNA synthesis modulation. Cell Death Dis..

[20] Glodzik D (2023). Detection of biallelic loss of DNA repair genes in formalin-fixed, paraffin-embedded tumor samples using a novel tumor-only sequencing panel.. J. Mol. Diagn..

[21] Edwards SL (2008). Resistance to therapy caused by intragenic deletion in *BRCA2*. Nature.

[22] Weigelt B (2017). Diverse *BRCA1* and *BRCA2* reversion mutations in circulating cell-free DNA of therapy-resistant breast or ovarian cancer. Clin. Cancer Res..

[23] Yap TA (2020). Phase I trial of first-in-class ATR inhibitor M6620 (VX-970) as monotherapy or in combination with carboplatin in patients with advanced solid tumors. J. Clin. Oncol..

[24] Dillon M (2019). A phase I study of ATR inhibitor, AZD6738, as monotherapy in advanced solid tumours (PATRIOT part A, B). Ann. Oncol..

[25] Yap TA (2022). Phase Ib expansion trial of the safety and efficacy of the oral ataxia telangiectasia and Rad3-related (ATR) inhibitor elimusertib in advanced solid tumors with DNA damage response (DDR) defects. Cancer Res..

[26] Cancer Genome Atlas Research Network. (2011). Integrated genomic analyses of ovarian carcinoma. Nature.

[27] Ceccaldi R (2015). A unique subset of epithelial ovarian cancers with platinum sensitivity and PARP inhibitor resistance. Cancer Res..

[28] Konstantinopoulos PA (2021). A replication stress biomarker is associated with response to gemcitabine versus combined gemcitabine and ATR inhibitor therapy in ovarian cancer. Nat. Commun..

[29] Konstantinopoulos PA, Lheureux S, Moore KN (2020). PARP inhibitors for ovarian cancer: current indications, future combinations, and novel assets in development to target DNA damage repair. Am. Soc. Clin. Oncol. Educ. Book.

[30] da Costa A (2023). Targeting replication stress in cancer therapy. Nat. Rev. Drug Discov..

[31] Zheng Z (2014). Anchored multiplex PCR for targeted next-generation sequencing. Nat. Med..

[32] Shen R, Seshan VE (2016). FACETS: allele-specific copy number and clonal heterogeneity analysis tool for high-throughput DNA sequencing. Nucleic Acids Res..

[33] Breast Cancer Association Consortium et al. Pathology of tumors associated with pathogenic germline variants in 9 breast cancer susceptibility genes. *JAMA Oncol.* **8**, e216744 (2022). 10.1001/jamaoncol.2021.6744 PMC8796069 35084436

[34] Weigelt B (2018). The landscape of somatic genetic alterations in breast cancers from ATM germline mutation carriers. J. Natl Cancer Inst..

[35] Renault AL (2018). Morphology and genomic hallmarks of breast tumours developed by ATM deleterious variant carriers. Breast Cancer Res..

[36] Honrado E (2005). Immunohistochemical expression of DNA repair proteins in familial breast cancer differentiate *BRCA2*-associated tumors. J. Clin. Oncol..

[37] Breast Cancer Association Consortium et al. Breast cancer risk genes—association analysis in more than 113,000 women. *N. Engl. J. Med.* **384**, 428–439 (2021). 10.1056/NEJMoa1913948 PMC7611105 33471991

[38] Hakem R (1996). The tumor suppressor gene *Brca1* is required for embryonic cellular proliferation in the mouse. Cell.

[39] Furuta S (2005). Depletion of BRCA1 impairs differentiation but enhances proliferation of mammary epithelial cells. Proc. Natl Acad. Sci. USA.

[40] Toledo LI (2013). ATR prohibits replication catastrophe by preventing global exhaustion of RPA. Cell.

[41] Kanu N (2015). SETD2 loss-of-function promotes renal cancer branched evolution through replication stress and impaired DNA repair. Oncogene.

[42] Pfister SX (2015). Inhibiting WEE1 selectively kills histone H3K36me3-deficient cancers by dNTP starvation. Cancer Cell.

[43] Toledo LI (2011). A cell-based screen identifies ATR inhibitors with synthetic lethal properties for cancer-associated mutations. Nat. Struct. Mol. Biol..

[44] Schoppy DW (2012). Oncogenic stress sensitizes murine cancers to hypomorphic suppression of ATR. J. Clin. Invest..

[45] Guerrero Llobet S (2022). An mRNA expression-based signature for oncogene-induced replication-stress. Oncogene.

[46] Sokol ES (2022). PARP inhibitor insensitivity to *BRCA1/2* monoallelic mutations in microsatellite instability-high cancers. JCO Precis. Oncol..

[47] de Bono J (2020). Olaparib for metastatic castration-resistant prostate cancer. N. Engl. J. Med..

[48] Kim ES (2022). Blood-based tumor mutational burden as a biomarker for atezolizumab in non-small cell lung cancer: the phase 2 B-F1RST trial. Nat. Med..

[49] Stadler JC (2022). Current and future clinical applications of ctDNA in immuno-oncology. Cancer Res..

[50] Oliveira KCS (2020). Current perspectives on circulating tumor DNA, precision medicine, and personalized clinical management of cancer. Mol. Cancer Res..

[51] Cescon DW, Bratman SV, Chan SM, Siu LL (2020). Circulating tumor DNA and liquid biopsy in oncology. Nat. Cancer.

[52] Wan JCM (2017). Liquid biopsies come of age: towards implementation of circulating tumour DNA. Nat. Rev. Cancer.

[53] Lord CJ, Ashworth A (2013). Mechanisms of resistance to therapies targeting BRCA-mutant cancers. Nat. Med..

[54] Domchek SM (2017). Reversion mutations with clinical use of PARP inhibitors: many genes, many versions. Cancer Discov..

[55] Plummer R (2022). A phase 1b study evaluating the safety and preliminary efficacy of berzosertib in combination with gemcitabine in patients with advanced non-small cell lung cancer. Lung Cancer.

[56] Coombs CC (2017). Therapy-related clonal hematopoiesis in patients with non-hematologic cancers is common and associated with adverse clinical outcomes. Cell Stem Cell.

[57] Kurnit KC (2018). Precision oncology decision support: current approaches and strategies for the future. Clin. Cancer Res..

[58] Yuan Y, Hess KR, Hilsenbeck SG, Gilbert MR (2016). Bayesian optimal interval design: a simple and well-performing design for phase I oncology trials. Clin. Cancer Res..

[59] Rosenthal R (2016). DeconstructSigs: delineating mutational processes in single tumors distinguishes DNA repair deficiencies and patterns of carcinoma evolution. Genome Biol..

[60] Alexandrov LB (2020). The repertoire of mutational signatures in human cancer. Nature.

[61] Buisson R, Boisvert JL, Benes CH, Zou L (2015). Distinct but concerted roles of ATR, DNA-PK, and Chk1 in countering replication stress during S phase. Mol. Cell.

