# Supplementary material for: Camonsertib in DNA damage response-deficient advanced solid tumors: phase 1 trial results
Source: Nat Med. 2023 Jun 5;29(6):1400–11. doi: 10.1038/s41591-023-02399-0 (PMC10287555; doi:10.1038/s41591-023-02399-0)
Supplement: Supplementary file 1 — Supplementary Tables 1–5 and Supplementary Figs. 1–7 [file 41591_2023_2399_MOESM1_ESM.pdf]

nature medicine

Article https://doi.org/10.1038/s41591-023-02399-0

Camonsertib in DNA damage response-deficient advanced solid tumors: phase 1 trial results

In the format provided by the authors and unedited

Supplementary Information for:

Camonsertib in DNA damage response-deficient advanced solid tumors: phase 1 trial results

Supplementary Tables 3

Supplementary Table 1 Pharmacokinetic parameters of camonsertib 4

Supplementary Table 2 Summary of anti-tumor activity by enrollment gene in efficacy population with dose >100 mg/day.. 7

Supplementary Table 3 Patients with discordant ctDNA molecular response and clinical outcomes... 8

Supplementary Table 4 Patients in which the enrollment alteration was determined to be from CHIP 9

Supplementary Table 5 Reversion alterations and clinical outcomes detected in patients treated with camonsertib in TRESR.. 10

Supplementary Figures 11

Supplementary Figure 1 Patient samples available for translational analysis. 12

Supplementary Figure 2 Plasma levels of camonsertib do not affect ΔQTcF. 13

Supplementary Figure 3 Food effect on the pharmacokinetics of camonsertib. 14

Supplementary Figure 4 Catalogue of Somatic Mutations in Cancer (COSMIC) Signatures of the two responding patients enrolling with monoallelic alterations. 15

Supplementary Figure 5 Number of alterations determined to be derived from CHIP. 16

Supplementary Figure 6 The ABCDs of diagnosing ATM-altered tumors. 17

Supplementary Figure 7 Case study of a patient with germline *BRCA1*-mutated breast cancer in which the detected polyclonal *BRCA1* reversion alterations decline with camonsertib treatment. 19

Supplementary Tables

Supplementary Table 1 Pharmacokinetic parameters of camonsertib

Cycle 1 Day 1											
**Dose, mg**	**Tau, hr**		**T_{max}, hr**	**C_{max}, μg/mL**	**C_{max}/dose, μg/mL/mg**	**AUC_{0-t}, hr*μg/mL**	**AUC_{0-t}/dose, hr*μg/mL/mg**	**AUC_{0-INF}, hr*μg/mL**	**AUC_{0-12}, hr*μg/mL**	**Half-life, hr**
5	24	*n*	1	1	1	1	1	-	1	1
		Min	1.17	-	-	-	-	-	-	-
		Median	1.17	-	-	-	-	-	-	-
		Max	1.17	-	-	-	-	-	-	-
		Geometric mean	-	0.5	0.098	2.1	0.427	2.3	1.8	5.87
		Geometric SD	-	-	-	-	-	-	-	-
		Geometric CV%	-	-	-	-	-	-	-	-
10	24	*n*	1	1	1	1	1	1	1	1
		Min	1	-	-	-	-	-	-	-
		Median	1	-	-	-	-	-	-	-
		Max	1	-	-	-	-	-	-	-
		Geometric mean	-	1.1	0.105	5.7	0.567	6.6	4.4	8.5
		Geometric SD	-	-	-	-	-	-	-	-
		Geometric CV%	-	-	-	-	-	-	-	-
20	24	*n*	2	2	2	2	2	1	2	1
		Min	0.97	-	-	-	-	-	-	-
		Median	10.71	-	-	-	-	-	-	-
		Max	20.45	-	-	-	-	-	-	-
		Geometric mean	-	0.8	0.04	4.8	0.239	3.1	2.8	4.18
		Geometric SD	-	1.1	1.071	1.9	1.919	-	1	-
		Geometric CV%	-	6.9	6.9	72.7	72.7	-	0.5	-
40	24	*n*	1	1	1	1	1	0	1	0
		Min	22.13	-	-	-	-	-	-	-
		Median	22.13	-	-	-	-	-	-	-
		Max	22.13	-	-	-	-	-	-	-
		Geometric mean	-	1.2	0.029	13.2	0.329	-	4.7	-
		Geometric SD	-	-	-	-	-	-	-	-
		Geometric CV%	-	-	-	-	-	-	-	-
80	24	*n*	1	1	1	1	1	1	1	1
		Min	1	-	-	-	-	-	-	-
		Median	1	-	-	-	-	-	-	-
		Max	1	-	-	-	-	-	-	-
		Geometric mean	-	5.7	0.071	20.3	0.254	21	18.7	4.08
		Geometric SD	-	-	-	-	-	-	-	-
		Geometric CV%	-	-	-	-	-	-	-	-
100	24	*n*	8	8	8	8	8	8	8	8
		Min	1	-	-	-	-	-	-	-
		Median	1.54	-	-	-	-	-	-	-
		Max	4	-	-	-	-	-	-	-
		Geometric mean	-	5.2	0.052	30.2	0.3	32.3	24.8	5.67
		Geometric SD	-	1.3	1.333	1.5	1.5	1.6	1.4	1.3
		Geometric CV%	-	29.4	29.4	44.2	44.2	50.4	37.1	26.69
120	24	*n*	31	31	31	31	31	30	31	30
		Min	0.5	-	-	-	-	-	-	-

		Median	2.03	-	-	-	-	-	-	-
		Max	4.15	-	-	-	-	-	-	-
		Geometric mean	-	5.3	0.044	29.5	0.246	31.3	23.9	6.21
		Geometric SD	-	1.5	1.519	1.7	1.687	1.7	1.6	1.34
		Geometric CV%	-	43.7	43.7	56.1	56.1	57.8	48.9	30.06
160	24	*n*	62	62	62	62	62	60	61	60
		Min	0.5	-	-	-	-	-	-	-
		Median	1.21	-	-	-	-	-	-	-
		Max	6.08	-	-	-	-	-	-	-
		Geometric mean	-	7.3	0.046	41	0.256	45.9	34.8	5.97
		Geometric SD	-	1.5	1.453	1.7	1.657	1.7	1.5	1.42
		Geometric CV%	-	38.7	38.7	53.9	53.9	58.7	44.2	36.29
200	24	*n*	5	5	5	5	5	5	5	5
		Min	0.5	-	-	-	-	-	-	-
		Median	1	-	-	-	-	-	-	-
		Max	1.75	-	-	-	-	-	-	-
		Geometric mean		9.5	0.047	54.6	0.273	58.9	44.7	6.31
		Geometric SD		1.4	1.423	1.2	1.228	1.2	1.2	1.13
		Geometric CV%		36.4	36.4	20.7	20.7	22.2	20.3	12.49
40	12	*n*	2	2	2	2	2	2	2	ND
		Min	1.92	-	-	-	-	-	-	-
		Median	1.96	-	-	-	-	-	-	-
		Max	2	-	-	-	-	-	-	-
		Geometric mean	-	3.8	0.095	40.1	1.003	152.6	23.4	-
		Geometric SD	-	1.2	1.158	1.7	1.677	1.8	1.6	-
		Geometric CV%	-	14.7	14.7	55.3	55.3	65.3	49.9	-
60	12	*n*	4	4	4	4	4	4	4	ND
		Min	1	-	-	-	-	-	-	-
		Median	1.55	-	-	-	-	-	-	-
		Max	2.32	-	-	-	-	-	-	-
		Geometric mean		3.3	0.055	26.6	0.444	66.1	16.9	-
		Geometric SD		1.4	1.449	1.5	1.507	1.8	1.5	-
		Geometric CV%		38.4	38.4	42.8	42.8	67.3	43.2	-
80	12	*n*	1	1	1	1	1	0	1	ND
		Min	2.17	-	-	-	-	-	-	-
		Median	2.17	-	-	-	-	-	-	-
		Max	2.17	-	-	-	-	-	-	-
		Geometric mean	-	3	0.038	42.1	0.527	-	21.4	-
		Geometric SD	-	-	-	-	-	-	-	-
		Geometric CV%	-	-	-	-	-	-	-	-

AUC, area under the concentration-time curve; C_{max}, maximum observed plasma concentration; CV, coefficient of variation; hr, hour; max, maximum; min, minimum; ND; not determined; SD, standard deviation; T_{max}, time to reach C_{max}.

Supplementary Table 2 Summary of anti-tumor activity by enrollment gene in efficacy population with dose >100 mg/day

	All patients (*N* = 99)	*ATM* (*n* = 34)	*BRCA1* (*n* = 23)	*BRCA2* (*n* = 14)	*SETD2* (*n* = 5)	*CDK12* (*n* = 7)	Others[a] (*n* = 16)
Best response by RECIST 1.1, n (%)							
Partial response (confirmed)	8 (8.1%)	2 (5.9%)	2 (8.7%)	1 (7.1%)	1 (20.0%)	1 (14.3%)	1 (6.3%)[b]
Partial response (unconfirmed)	2 (2.0%)	0	2 (8.7%)	0	0	0	0
Best response by tumor marker, n (%)							
PCWG3 PSA response (confirmed)	2 (2.0%)	2 (5.9%)	0	0	0	0	0
GCIG CA-125 response (confirmed)	1 (1.0%)	0	0	0	0	0	1 (6.3%)[b]
Molecular response (ctDNA ≥50% reduction)							
Evaluable ctDNA for monitoring, n	63	23	18	10	0	3	9
Molecular response, n/m (%)	27/63 (42.9%)	9/23 (39.1%)	9/18 (50.0%)	6/10 (60.0%)	0	1/3 (33.3%)	2/9 (22.2%)[c]
Response by RECIST or tumor marker (response rate)	13 (13.1%)	4 (11.8%)	4 (17.4%)	1 (7.1%)	1 (20.0%)	1 (14.3%)	2 (12.5%)
Clinical benefit rate (response or DOT ≥16 weeks w/o PD)	43 (43.4%)	15 (44.1%)	11 (47.8%)	5 (35.7%)	4 (80.0%)	2 (28.6%)	6 (37.5%)
Median progression-free survival, weeks	15.1	17.3	12.3	15.3	37.0	11.6	12.1

Data cut-off date = March 03, 2022.

[a]Includes *CHEK2* (*n* = 2), *NBN* (*n* = 4), *PALB2* (*n* = 4), *RAD51C* (*n* = 3), and *RNASEH2* (*n* = 3). [b]Both the RECIST 1.1 confirmed cPR and GCIG CA-125 confirmed response were from patients with *RAD51C* alterations. The patient with the *RAD51C* alteration and cPR later went on to achieve a CR following resolution of the non-target lesion.

[c]Patients with molecular responses had *PALB2* and *RAD51C* alterations.

ctDNA, circulating tumor DNA; cPR, confirmed partial response; CR, confirmed response; DOT, duration of treatment; GCIG, Gynecologic Cancer Intergroup; PCWG3, Prostate Cancer Working Group 3; PD, progressive disease; PSA, prostate-specific antigen; RECIST, Response Evaluation Criteria in Solid Tumors; w/o, without.

Supplementary Table 3 Patients with discordant ctDNA molecular response and clinical outcomes

Patient	Tumor	Gene	RECIST 1.1 or PSA/CA125 response[a]	Best ctDNA mVAF change[b]	Relevant observations	Hypothesized reason for discordance
69F	Breast	g*BRCA1*	SD, no clinical benefit	-89% (molecular response)	Starting mVAF was below 1%. PD at 12 wks was due to new brain lesion (camonsertib is not brain-penetrant); extra-cranial tumor lesions had reduced in size (-27% at 1st scan, -19% at 2nd scan)	Difficult to monitor due to low starting mVAF
71M	NHSCC	s*BRCA1*	PR	-15% (stable)	Starting mVAF was below 1%.	Difficult to monitor due to low starting mVAF
64F	Ovarian	*BRCA1*	SD, clinical benefit	+53% (rising)	Starting mVAF was below 1%. Patient was on treatment >16 wks with slow progression. Patient had dose reduction at cycle 2. Tumor lesions increased in size, with PD at the 3rd tumor assessment (18 wks)	Difficult to monitor due to low starting mVAF
74F	Ovarian	g*BRCA1*	PR	>100% (rising)	Starting mVAF was below 1%. RECIST 1.1 PR with a discordant increase in ctDNA mVAF. A *PIK3CA* alteration was observed to increase from 0.7 to 1.5% VAF at cycle 3 day 1	Difficult to monitor due to low starting mVAF
71M	Colorectal	s*ATM*	SD, clinical benefit	>100% (rising)	Starting mVAF was below 1%. Was on trial for 16 wks with tumor lesions increasing during this time, with PD at the 3rd tumor assessment	Difficult to monitor due to low starting mVAF
71M	Pancreatic	g*ATM*	SD, no clinical benefit	-94% (molecular response)	Aggressive tumor. Somatic variants, including *KRAS* G12D declined >90% on-therapy at week 3. ctDNA rebounded quickly thereafter, concurrent with treatment discontinuation	Heterogenous or aggressive tumor
58F	Breast	g*BRCA2*	SD, no clinical benefit	-90% (molecular response)	Tumor heterogeneity. PD was due to new liver mets, but overall target lesions had decreased vs baseline. ctDNA reduction was based on *ESR1* mutation that could have been subclonal	Heterogenous or aggressive tumor
49F	Breast	g*BRCA1*	PD	-65% (molecular response)	Aggressive tumor. Patient had RECIST 1.1 PD at 1st scan; heavily pretreated (10 lines); possible progression between the baseline scan and start of therapy as baseline scan was done 3–4 weeks prior to trial start. Multiple *ESR1* mutations suggestive of polyclonality. Majority of ctDNA decrease driven by a single mutation	Heterogenous or aggressive tumor
71F	Pancreatic	g*ATM*	SD, no clinical benefit	-95% (molecular response)	Dose reduction at wk 8 due to G2 neutrophil decrease and anemia. Patient had RECIST 1.1 SD (target lesions -4%) at 2nd scan (of wk 10), but was discontinued from treatment due to clinical progression. ctDNA reduction was based on a *KRAS* mutation that could have been subclonal	Sub-optimal exposure and tumor heterogeneity
49F	Ovarian	s*BRCA2*	PD	-94% (molecular response)	Patient had prolonged dose hold (for 4 wks) after only 1 wk of treatment. Patient had progressive disease at 1st restaging scan, but patient only had 1 wk of treatment by the time of the scan	Sub-optimal drug exposure
63M	Prostate	g*BRCA2*	SD, no clinical benefit	-88% (molecular response)	Patient had Gr4 thrombocytopenia, febrile neutropenia, and anemia on cycle 1 day 15. Resumed cycle 2 at reduced dose after 3-wk dose hold	Sub-optimal drug exposure
74M	Prostate	g*ATM*	PSA response	-16% (stable)	Prostate cancer with non-measurable disease	Unknown
70F	Ovarian	*BRCA2*	SD, clinical benefit	-1% (stable)	No observed decrease in target lesion	Unknown

72F	Endometrial	*BRCA1*	PD	-64% (molecular response)	Dose hold on cycle 1 day 8 for 1 wk due to unrelated SAE; early scan at wk 4 showed PD due to nontarget lesion progression (12% increase in target lesions)	Unknown

[a]Clinical benefit was defined as a patient having a treatment duration of at least 16 weeks (without evidence of progression) and/or having a RECIST 1.1 or tumor marker response.

[b]ctDNA monitoring: at least one somatic variant above 1% VAF and mean VAF above 0.5% at baseline for the patient to be evaluable.

CA125, cancer antigen 125; ctDNA, circulating tumor DNA; F, female; g, germline; M, male; mets, metastases; mVAF; mean VAF; PD, progressive disease; PR, partial response; PSA, prostate-specific antigen; RECIST, Response Evaluation Criteria in Solid Tumors; s, somatic; SAE, serious adverse event; SD, stable disease; VAF, variant allele frequency; wk, week.

Supplementary Table 4 Patients in which the enrollment alteration was determined to be from CHIP

Tumor type	Enrollment test	Enrollment alteration	Local result (VAF), %	Central PBMC (VAF)	Central ctDNA (VAF)	Central tumor biopsy	Central determination	Clinical summary
Breast	Liquid	*ATM* p.G2891D	10	10%	11%	ND	CHIP	Treated for 15 weeks; continually increasing CA 15-3, without any decrease in tumor volume
CCA	Tissue	*ATM* c.2921+1G>A	N/A	52%	50%	7% (low tumor purity)	CHIP	Treated[a] for 6 weeks; disease progression at first scan
Prostate	Liquid	*ATM* p.R3008H	8	23%	10%	11% (low tumor purity)	CHIP	Treated for 7 weeks; discontinued due to clinical progression, increasing target lesion and PSA

[a]100 mg once daily.

CA 15-3, cancer antigen 15-3; CCA, cholangiocarcinoma; CHIP, clonal hematopoiesis of indeterminate potential; ctDNA, circulating tumor DNA; N/A, not applicable; ND, not detected; PBMC, peripheral blood mononuclear cells; PSA, prostate-specific antigen; VAF, variant allele frequency.

Supplementary Table 5 Reversion alterations and clinical outcomes detected in patients treated with camonsertib in TRESR

Diagnosis	Prior PARPi or platinum	Gene	Zygosity	Primary cDNA	Primary amino acid	Reversion detection platform	First timepoint detected	Number of unique reversions	Clinical outcome	Clinical benefit
Breast	Olaparib	s*BRCA1*	Biallelic	c.1717_1718insT	p.S573fs*13	Tissue and liquid	Baseline	6	SD (off tx, 29w)	Y
Breast	Carboplatin Rucaparib Cisplatin	g*BRCA1*	Biallelic	c.4127_4128insT	p.S1377fs*4	Tissue	Baseline	1	SD (off tx, 19w)	Y
Breast	Olaparib	g*BRCA1*	Biallelic	c.5266dupC	p.Q1756fs	Liquid	Baseline	11	PD (off tx 6 w)	N
Breast	Olaparib	g*BRCA2*	Unknown	c.6092_6095dup	p.I2033fs*17	Tissue	Baseline	1	SD (off tx 4 w)	N
Ovarian	Carboplatin Olaparib Rucaparib	g*BRCA1*	Biallelic	c.427G>T	p.E143*	Tissue	Progression[a]	1	PR (off tx 48w)	Y
Ovarian	Carboplatin, 2X-121	g*RAD51C*	Unknown	c.701C>G	p.S234*	Liquid	Baseline	1	SD (off tx 19w)	Y
Pancreatic	Niraparib	s*BRCA2*	Biallelic	c.2957_2958insA	p.N986fs*2	Tissue and liquid	Baseline	1	PD (off tx 3w, early restaging)	N
Pancreatic	Oxaliplatin	g*BRCA2*	Biallelic	c.6052_6053delAG	p.S2018fs	Tissue	Baseline	1	SD (off tx 26)	Y
Pancreatic	Oxaliplatin	s*NBN*	Biallelic	c.1146del	p.E383fs*21	Tissue	Baseline	1	SD (off tx 12w)	N
Prostate	Olaparib	g*PALB2*	Monoallelic	c.3323del	p.Y1108fs*16	Tissue	Baseline	1	PD (off tx, 6w)	N

[a]Baseline tissue not available.

cDNA, circulating DNA; g, germline; N, no; PARPi, poly adenosine diphosphate-ribose polymerase inhibitor; PD, progressive disease; SD, stable disease; s, somatic; tx, treatment; w, weeks; Y, yes.

Supplementary Figures

Supplementary Figure 1 Patient samples available for translational analysis.

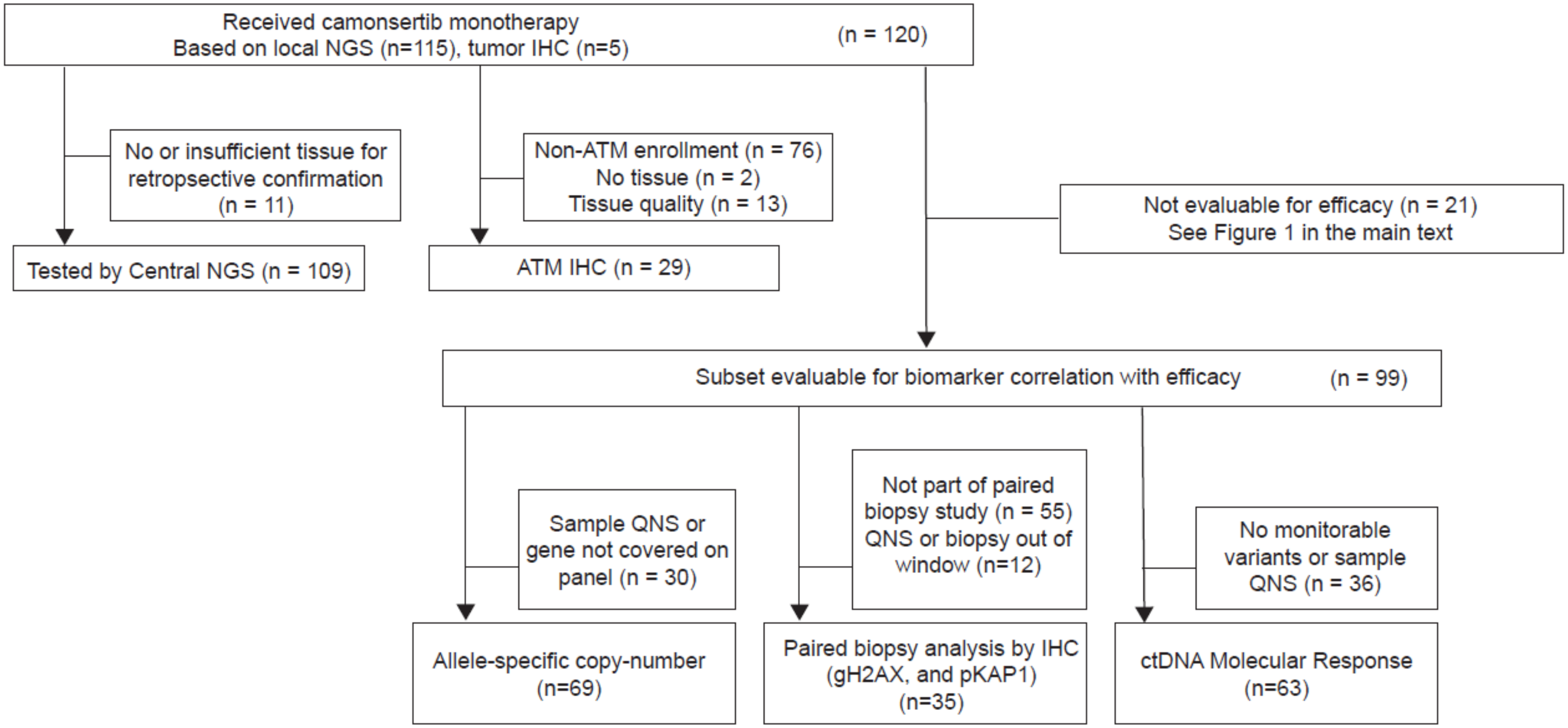

ctDNA, circulating tumor DNA; IHC, immunohistochemistry; NGS, next-generation sequencing; QNS, quality not sufficient.

Supplementary Figure 2 Plasma levels of camonsertib do not affect ΔQTcF. The mean ΔQTcF versus time is close to 0 for any concentration measurements and the 90% confidence interval upper bound is less than 10 ms at any time, suggesting no drug influence on ΔQTcF.

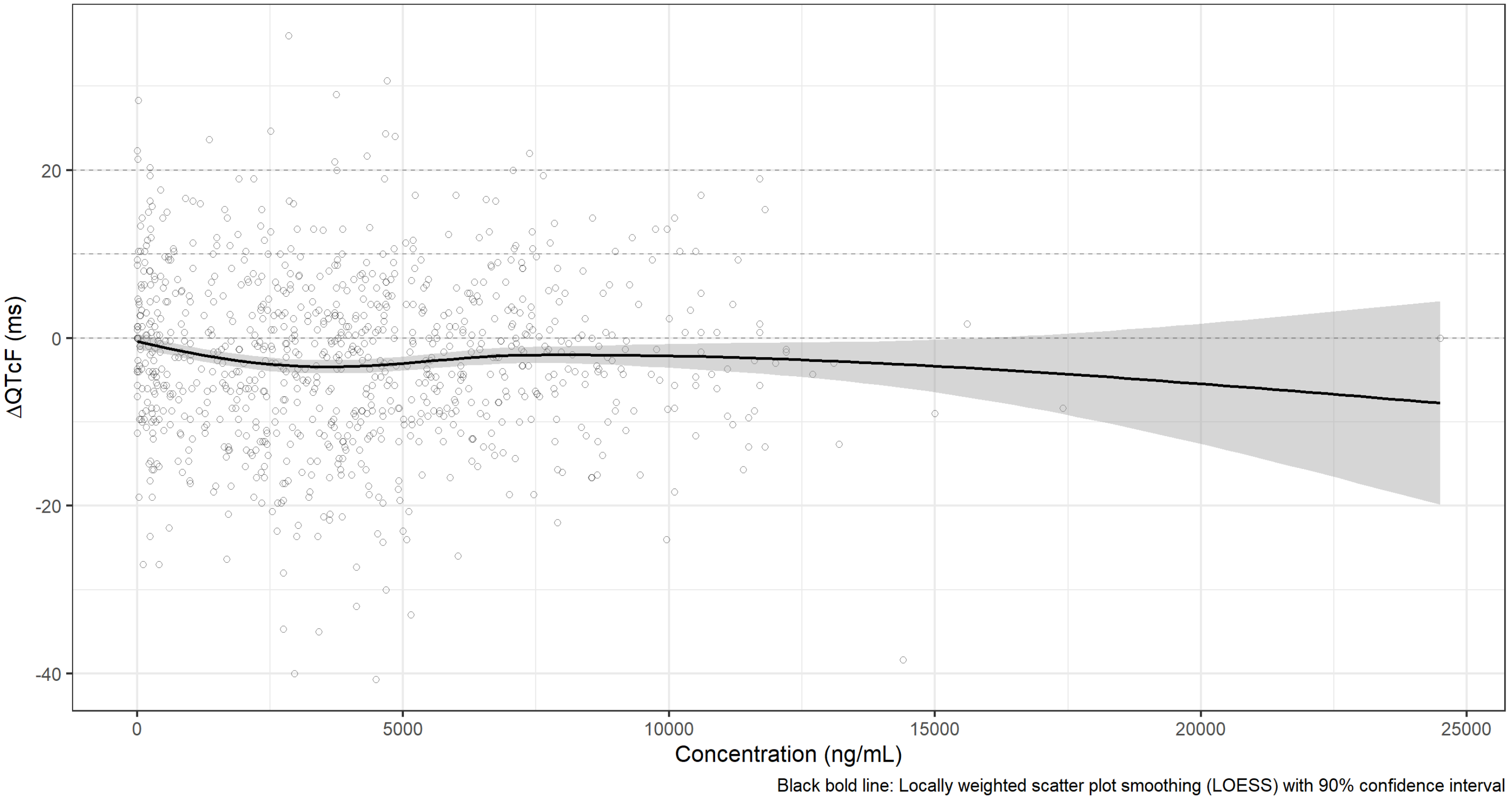

QTcF, Fridericia formula for corrected QT interval.

Supplementary Figure 3 Food effect on the pharmacokinetics of camonsertib. Geometric mean plasma concentrations of camonsertib when administered with either a high fat/high calorie mean (yellow circles) or in the fasted state (red crosses) are plotted at dose levels of 100 mg QD (*n* = 1 patient), 120 mg QD (*n* = 8 patients), or 160 mg QD (*n* = 3 patients). Error bars represent geometric standard deviation. A delay in T_{max} and a reduction is in C_{max} is observed when camonsertib is administered with a high fat/high calorie meal; however, there is no change in AUC and, importantly, the plasma levels required for efficacy. The red-dashed line represents the pre-clinical in vivo tumor pCHK1 IC_{80}. Note that not every patient was evaluated at every timepoint.

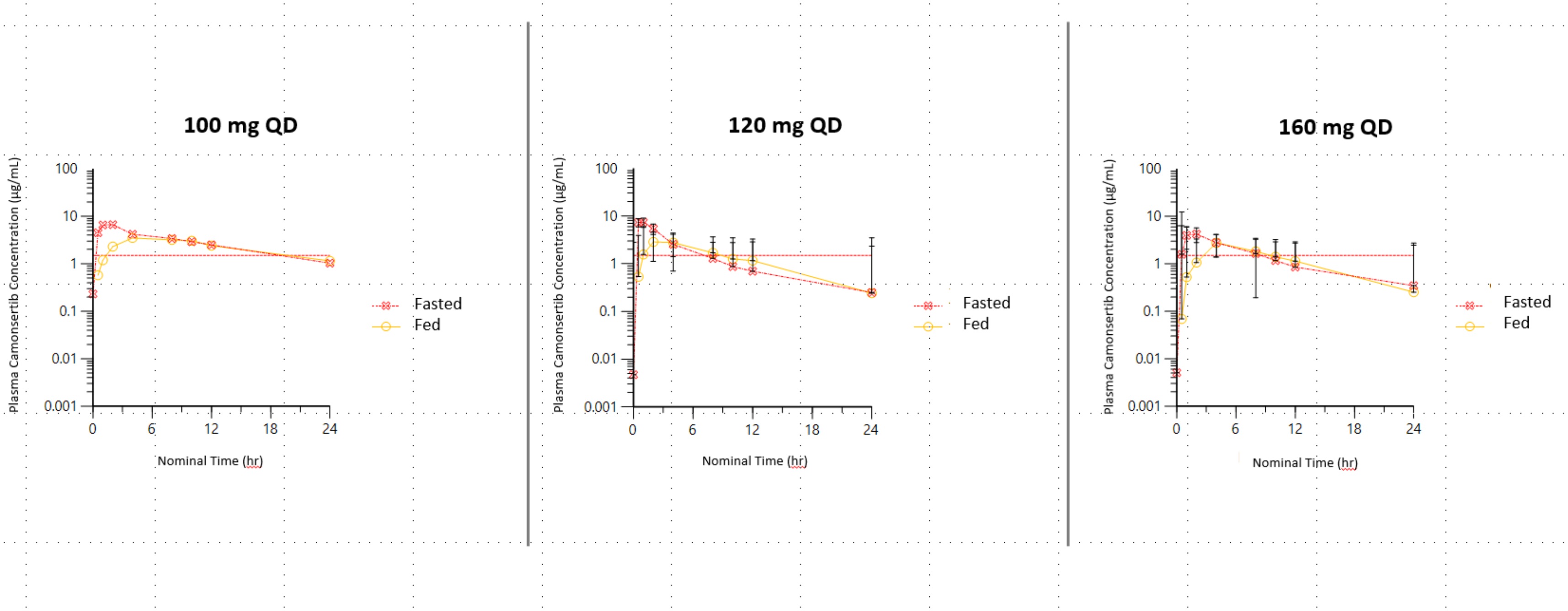

AUC, area under the concentration-time curve; C_{max}, maximum observed plasma concentration; IC_{80}, 80% inhibitory concentration; QD, once daily; T_{max}, time to reach C_{max}.

Supplementary Figure 4 Catalogue of Somatic Mutations in Cancer (COSMIC) signatures of the two responding patients enrolling with monoallelic alterations. (Top) Melanoma tumor with a monoallelic somatic *BRCA2* alteration and UV light signature. (Bottom) Head and neck squamous cell carcinoma tumor with monoallelic *BRCA1* alteration and APOBEC signature.

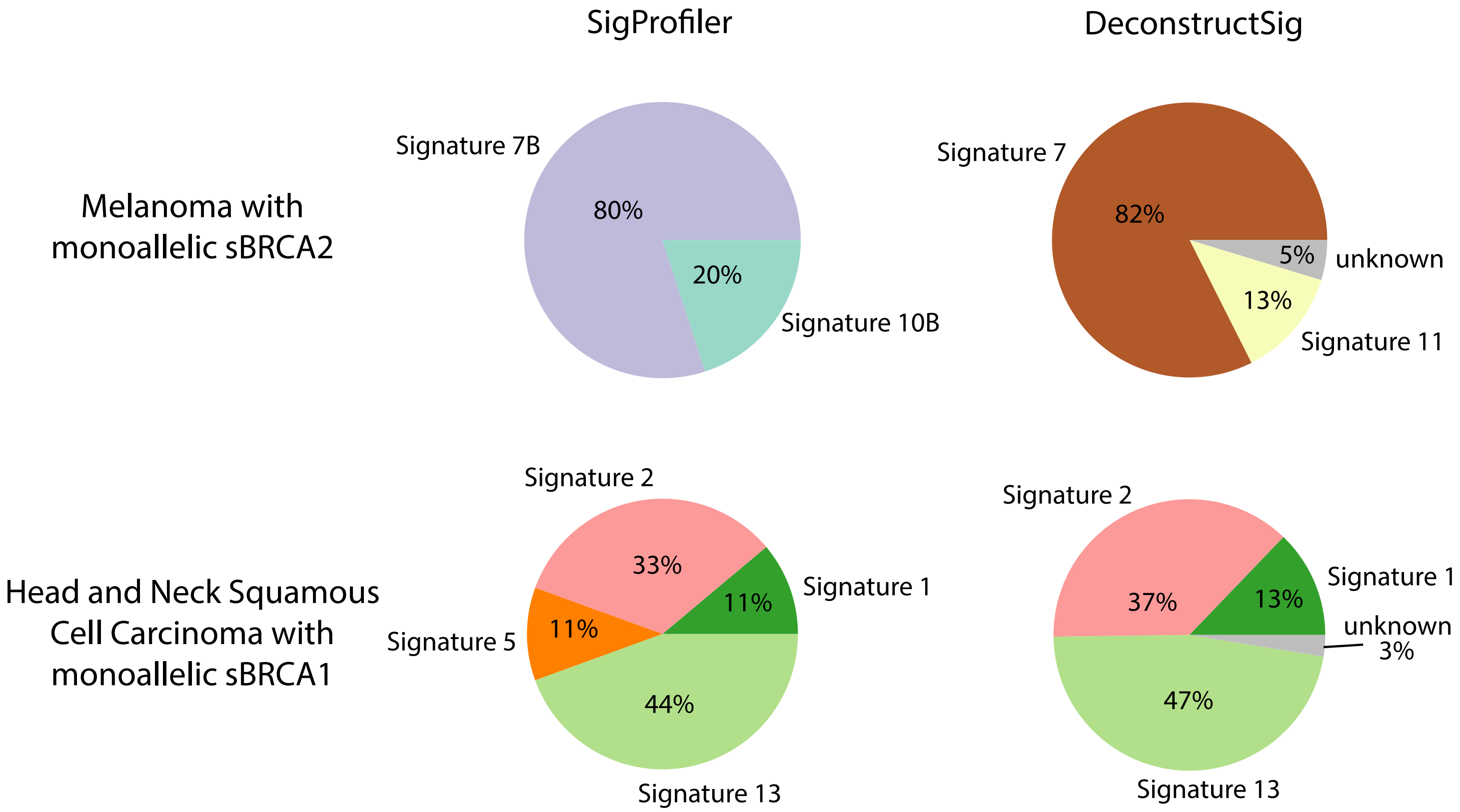

APOBEC, apolipoprotein B mRNA-editing enzyme catalytic polypeptide; s, somatic; UV, ultraviolet.

Supplementary Figure 5 Number of alterations determined to be derived from CHIP.

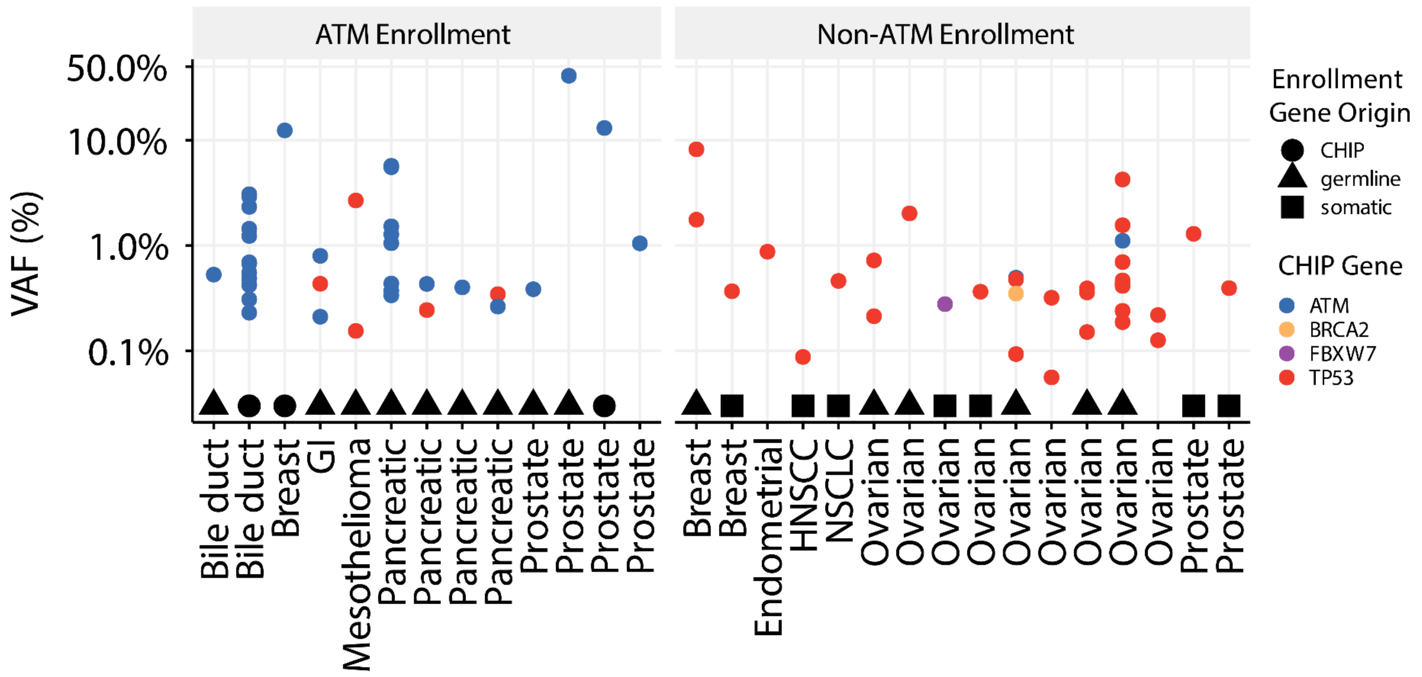

CHIP, clonal hematopoiesis of indeterminate potential; GI, gastrointestinal; HNSCC, head and neck squamous cell carcinoma; NSCLC, non-small cell lung cancer; VAF, variant allele frequency.

Supplementary Figure 6 The ABCDs of diagnosing *ATM*-altered tumors.

**Annotate:** *Mandate central variant review for ATM LOF (applies to other biomarkers as well)*
**Biallelic:** A*nalyze allelic status to identify the patients with biallelic ATM LOF (as opposed to monoallelic ATM LOF)*
**CHIP:** *Analyze PBMCs to ensure the variant is not derived from the bone marrow as part of clonal hematopoiesis*
**Do the IHC:** *Examine ATM protein expression in the context of genomic results*

IHC, immunohistochemistry; LOF, loss of function; PBMCs, peripheral blood mononuclear cells.

Supplementary Figure 7 Case study of a patient with germline *BRCA1*-mutated breast cancer in which the detected polyclonal *BRCA1* reversion alterations decline with camonsertib treatment. The patient had a dose hold at week 3, indicated by the black bar, and stopped treatment at week 6 due to an unrelated adverse event.

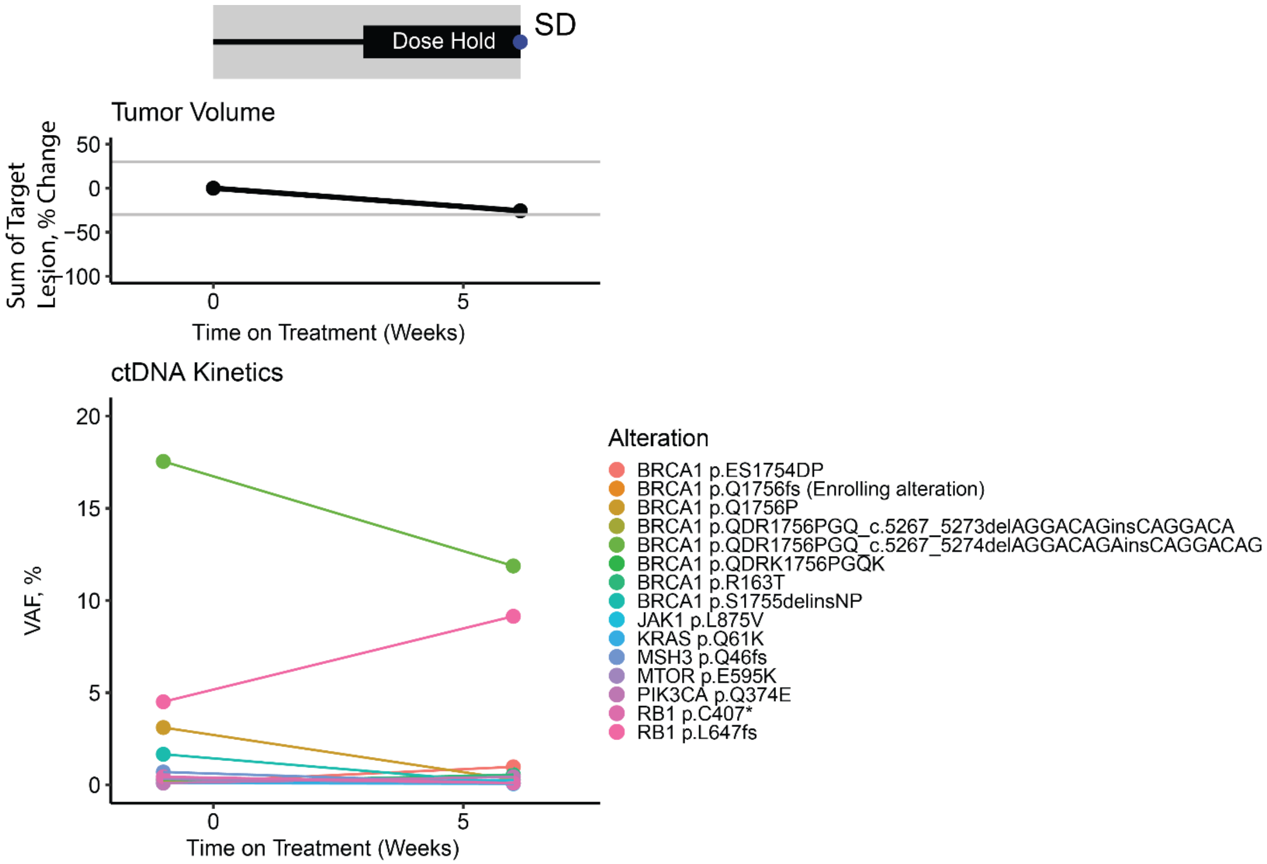

ctDNA, circulating tumor DNA; SD, stable disease; VAF, variant allele frequency.
