# Supplementary material for: Camonsertib in DNA damage response-deficient advanced solid tumors: phase 1 trial results
Source: Nat Med. 2023 Jun 5;29(6):1400–11. doi: 10.1038/s41591-023-02399-0 (PMC10287555; doi:10.1038/s41591-023-02399-0)
Supplement: Supplementary file 2 — Reporting Summary [file 41591_2023_2399_MOESM2_ESM.pdf]

nature portfolio

Corresponding author(s): Timothy Yap, MBBS, PhD, FRCP

Last updated by author(s): May 9, 2023

Reporting Summary

Nature Portfolio wishes to improve the reproducibility of the work that we publish. This form provides structure for consistency and transparency in reporting. For further information on Nature Portfolio policies, see our Editorial Policies and the Editorial Policy Checklist.

Statistics

For all statistical analyses, confirm that the following items are present in the figure legend, table legend, main text, or Methods section.

n/a	Confirmed	
☐	☒	The exact sample size (*n*) for each experimental group/condition, given as a discrete number and unit of measurement
☐	☒	A statement on whether measurements were taken from distinct samples or whether the same sample was measured repeatedly
☐	☒	The statistical test(s) used AND whether they are one- or two-sided<br>*Only common tests should be described solely by name; describe more complex techniques in the Methods section.*
☐	☒	A description of all covariates tested
☐	☒	A description of any assumptions or corrections, such as tests of normality and adjustment for multiple comparisons
☐	☒	A full description of the statistical parameters including central tendency (e.g. means) or other basic estimates (e.g. regression coefficient) AND variation (e.g. standard deviation) or associated estimates of uncertainty (e.g. confidence intervals)
☐	☒	For null hypothesis testing, the test statistic (e.g. *F*, *t*, *r*) with confidence intervals, effect sizes, degrees of freedom and *P* value noted<br>*Give P values as exact values whenever suitable.*
☒	☐	For Bayesian analysis, information on the choice of priors and Markov chain Monte Carlo settings
☒	☐	For hierarchical and complex designs, identification of the appropriate level for tests and full reporting of outcomes
☒	☐	Estimates of effect sizes (e.g. Cohen's *d*, Pearson's *r*), indicating how they were calculated

Our web collection on statistics for biologists contains articles on many of the points above.

Software and code

Policy information about availability of computer code

Data collection	SAS version 9.4M7; Alamut Batch database version 1.5-2020.11.25.
Data analysis	SAS version 9.4M7, Base R version 4.0; Phoenix v8.3.3.33; LoFreq v2.1.1, Freebayes v0.9.9; Archer Analysis Platform v6.2.8; Vcftools v0.1.11.

For manuscripts utilizing custom algorithms or software that are central to the research but not yet described in published literature, software must be made available to editors and reviewers. We strongly encourage code deposition in a community repository (e.g. GitHub). See the Nature Portfolio guidelines for submitting code & software for further information.

Data

Policy information about availability of data

All manuscripts must include a data availability statement. This statement should provide the following information, where applicable:

- Accession codes, unique identifiers, or web links for publicly available datasets
- A description of any restrictions on data availability
- For clinical datasets or third party data, please ensure that the statement adheres to our policy

To minimize the risk of patient re-identification, data will only be shared upon reasonable request. For eligible studies, qualified researchers may request access to individual patient-level clinical data through a data request platform. At the time of writing, this request platform is Vivli (https://vivli.org/ourmember/roche/). Datasets can be requested 18 months after a clinical study report has been completed and, as appropriate, once the regulatory review of the indication or drug has completed. Access to patient-level data from this trial can be requested and will be assessed by an independent review panel, which decides whether the data will

be provided. Once approved, the data are available for up to 24 months. For up-to-date details on Roche's Global Policy on the Sharing of Clinical Information and how to request access to related clinical study documents, see https://go.roche.com/data_sharing. Anonymized records for individual patients across more than one data source external to Roche cannot, and should not, be linked owing to a potential increase in risk of patient re-identification.

Research involving human participants, their data, or biological material

Policy information about studies with human participants or human data. See also policy information about sex, gender (identity/presentation), and sexual orientation and race, ethnicity and racism.

Reporting on sex and gender	In Table 1 we provide the breakdown of patients by sex (at birth) as reported to the patient study site. The primary aim of the study was to assess safety and tolerability of camonsertib in patients with DNA damage response-deficient solid tumors. The efficacy of ATR inhibition is thought to be dependent on the genetic profile (or the resulting DNA damage response functions) of the patient tumor. However, there is no evidence to suggest that the efficacy of camonsertib will be affected by sex or gender. Patients were therefore enrolled to the study and endpoints were assessed regardless of sex and gender.
Reporting on race, ethnicity, or other socially relevant groupings	We did not include any race or ethnicity information in any of the data reported on in this manuscript. There is no evidence to suggest that the efficacy of camonsertib will be affected by race/ethnicity. Patients were therefore enrolled to the study and endpoints were assessed regardless of race/ethnicity.
Population characteristics	The population included in our study was as specified in the inclusion criteria. The patient characteristics and breakdown of tumor types/genotypes of the population are indicated in Table 1.
Recruitment	Patients were recruited by study sites according to their meeting of study inclusion/exclusion criteria, without any bias.
Ethics oversight	The protocol was approved by the Institutional Review Board or ethics committee at each participating institution, namely: The University of Texas MD Anderson Cancer Center, Houston, TX, USA; The Sarah Cannon Research Institute UK, London, UK; The Dana-Farber Cancer Institute, Boston, MA, USA; The Sarah Cannon Research Institute/Tennessee Oncology, Nashville, TN, USA; The University Hospital of Copenhagen, Copenhagen, Denmark; The Princess Margaret Cancer Centre, Toronto, Canada; Duke University, Durham, NC, USA; Rhode Island Hospital, Providence, RI, USA; The Northern Centre for Cancer Care, Newcastle-upon-Tyne, UK; The Massachusetts General Hospital Cancer Center, Boston, MA, USA; The Memorial Sloan Kettering Cancer Center, New York, NY, USA; and The Christie Foundation, Manchester, UK.

Note that full information on the approval of the study protocol must also be provided in the manuscript.

Field-specific reporting

Please select the one below that is the best fit for your research. If you are not sure, read the appropriate sections before making your selection.

☒ Life sciences ☐ Behavioural & social sciences ☐ Ecological, evolutionary & environmental sciences

For a reference copy of the document with all sections, see nature.com/documents/nr-reporting-summary-flat.pdf

Life sciences study design

All studies must disclose on these points even when the disclosure is negative.

Sample size	Patient dispositions and n numbers for each stage of the study are shown in Figure 1d.
Data exclusions	Patient exclusion criteria and data filtering processes are described in the methods. All patients in Module 1 dose-finding cohorts were included in analyses and all main study objectives were reported on.
Replication	The number of patients included in each assessment is shown in the tables and figures. Human tissue and blood were analyzed in single replicates due to sample availability with validated assays.
Randomization	All 120 patients in this non-randomized study were allocated to camonsertib monotherapy as part of TRESR. Patients were subgrouped for planned exploratory endpoint assessments based on tumor type, immunohistochemical analyses, or molecular profiling (genotype, allelic status, or ctDNA analysis).
Blinding	The primary aim of the study was to assess safety and tolerability of camonsertib in patients with DNA damage response-deficient solid tumors. No blinding was performed in this open-label phase 1 study.

Reporting for specific materials, systems and methods

We require information from authors about some types of materials, experimental systems and methods used in many studies. Here, indicate whether each material, system or method listed is relevant to your study. If you are not sure if a list item applies to your research, read the appropriate section before selecting a response.

Materials & experimental systems

n/a	Involved in the study
☐	☒ Antibodies
☒	☐ Eukaryotic cell lines
☒	☐ Palaeontology and archaeology
☒	☐ Animals and other organisms
☐	☒ Clinical data
☒	☐ Dual use research of concern
☒	☐ Plants

Methods

n/a	Involved in the study
☒	☐ ChIP-seq
☒	☐ Flow cytometry
☒	☐ MRI-based neuroimaging

Antibodies

Antibodies used	Anti-phospho-histone H2A.X (Ser139) (20E3) (Anti-gH2Ax; Cell Signaling Technology, #9718. 1:1000 dilution).<br>Anti-ATM, clone Y170 (Abcam, ab32420. 1:250 dilution).<br>Anti-KAP1 (phospho S824) [BL-246-7B5] (Abcam, ab243870. 1:600 dilution).
Validation	Anti-phospho-histone H2A.X (Ser139) is validated for immunohistochemistry on paraffin-embedded human ovarian clear cell carcinoma. See https://www.cellsignal.com/products/primary-antibodies/phospho-histone-h2a-x-ser139-20e3-rabbit-mab/9718 for more details.<br>Anti-ATM is validated for immunohistochemistry on formaldehyde-fixed human serous ovarian tumor tissue and paraffin-embedded human breast cancer tissue. See https://www.abcam.com/products/primary-antibodies/atm-antibody-y170-ab32420.html for more details.<br>Anti-KAP1 is validated for immunohistochemistry on formalin-fixed, paraffin-embedded human lung carcinoma tissue. See https://www.abcam.com/products/primary-antibodies/kap1-phospho-s824-antibody-bl-246-7b5-ab243870.html for more details.

Clinical data

Policy information about clinical studies

All manuscripts should comply with the ICMJE guidelines for publication of clinical research and a completed CONSORT checklist must be included with all submissions.

Clinical trial registration	Clinicaltrials.gov NCT04497116
Study protocol	Attached as part of the submission
Data collection	Patients were enrolled between July 27, 2020 through November 1, 2021 and patient data was collected through March 22, 2022. Clinical data were collected at The University of Texas MD Anderson Cancer Center, Houston, TX, USA; The Sarah Cannon Research Institute UK, London, UK; The Dana-Farber Cancer Institute, Boston, MA, USA; The Sarah Cannon Research Institute/Tennessee Oncology, Nashville, TN, USA; The University Hospital of Copenhagen, Copenhagen, Denmark; The Princess Margaret Cancer Centre, Toronto, Canada; Duke University, Durham, NC, USA; Rhode Island Hospital, Providence, RI, USA; The Northern Centre for Cancer Care, Newcastle-upon-Tyne, UK; The Massachusetts General Hospital Cancer Center, Boston, MA, USA; The Memorial Sloan Kettering Cancer Center, New York, NY, USA; and The Christie Foundation, Manchester, UK.
Outcomes	Tolerability and safety of camonsertib was evaluated by assessment of adverse events, treatment-emergent adverse events, serious adverse events, dose-limiting toxicity, concomitant medications and procedures, physical exams, vital sign measurements, clinical safety laboratory evaluations (hematology, chemistry, and urinalysis), ECOG Performance Status scores, and electrocardiograms. The exploratory efficacy endpoint was assessment of antitumor activity by overall response rate, duration of treatment, clinical benefit rate, progression-free survival, and overall survival. Camonsertib plasma levels from cycle 1, day 1 of camonsertib were quantified using a validated liquid chromatography tandem-mass spectrometry (LC-MS/MS) method.
